# Developing *Emericellopsis* sp. XJ1056 as a versatile fungal platform for high-yield biosynthesis of nonribosomal peptides: a case study on beauvericin

**DOI:** 10.3389/fmicb.2026.1805553

**Published:** 2026-04-16

**Authors:** Yutong Ai, Kainan Song, Dongliang Xiao, Qun Yue, Chen Wang, Linan Xie, Liwen Zhang, Yuquan Xu

**Affiliations:** 1 National Key Laboratory of Agricultural Microbiology, Biotechnology Research Institute, Chinese Academy of Agricultural Sciences, Beijing, China; 2 Zhongyuan Research Center, Chinese Academy of Agricultural Sciences, Xinxiang, China; 3 College of Life and Health, Dalian University, Dalian, China

**Keywords:** beauvericin, cell factory, filamentous fungus, heterologous expression, nonribosomal peptide synthetase

## Abstract

**Introduction:**

Beauvericin is a cyclodepsipeptide with insecticidal, antitumor, and antimicrobial activities, yet its application is limited by low yields. Filamentous fungi represent promising cell factories for complex natural products, but constraints in genetic manipulation and productivity hinder their widespread use.

**Methods:**

In this study, we developed a high-efficiency fungal cell factory for the *de novo* biosynthesis of beauvericin. The filamentous fungus *Emericellopsis* sp. XJ1056, which natively produces high levels of the peptidyl product antiamoebins (5.2 g/kg), was selected as the host due to its exceptional peptide synthetic capacity. A robust genetic toolkit was established, including the construction of a *Δku70*strain to enhance homologous recombination efficiency (from 9.4% to 50–73.68%). Using *Δku70* as the chassis, we integrated the ∼10-kb beauvericin synthetase gene (*bbBeas*) via multiplexed homologous recombination, along with *kivr* (encoding 2-ketoisovalerate reductase) under the native *helA* promoter to supply the precursor D-hydroxyisovaleric acid (D-Hiv). Fermentation conditions were optimized, and strategies including split expression and codon optimization were evaluated.

**Results:**

The engineered strain *Δku70*-*bbBeas-kivr* synthesized beauvericin without precursor feeding. Optimal production (663.40 mg/kg) was achieved using rice supplemented with wheat bran as the solid medium. While split expression did not further enhance yield, codon optimization *(opbbBeas)* significantly increased transcriptional efficiency, raising beauvericin production to 921.24 mg/kg dry weight—surpassing the previously reported maximum (98.56 mg/L) in *Beauveria bassiana*.

**Discussion:**

This study achieves the highest beauvericin yield reported to date and establishes a generalizable platform for the heterologous production of diverse nonribosomal peptides (NRPs), with broad implications for sustainable biomanufacturing and natural product discovery.

## Graphical Abstract

**Figure:**
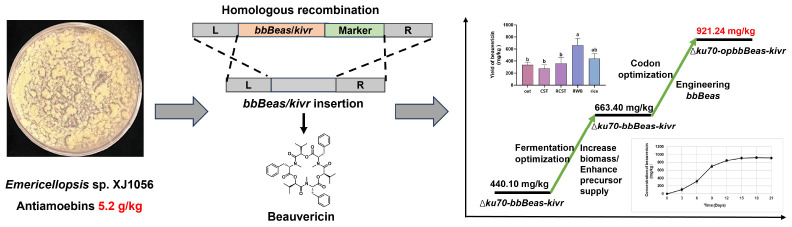
Schematic graph illustrating the development of *Emericellopsis* sp. XJ1056 as a versatile fungal platform for high-yield biosynthesis of nonribosomal peptides. A filamentous fungus (*Emericellopsis* sp. XJ1056) was engineered through a robust genetic toolkit (including *Δku70* chassis, multiplexed homologous recombination, and codon optimization) and fermentation optimization to achieve *de novo* biosynthesis of beauvericin at 921.24 mg/kg, establishing a generalizable platform for nonribosomal peptide production.

## Introduction

**FIGURE 1:**
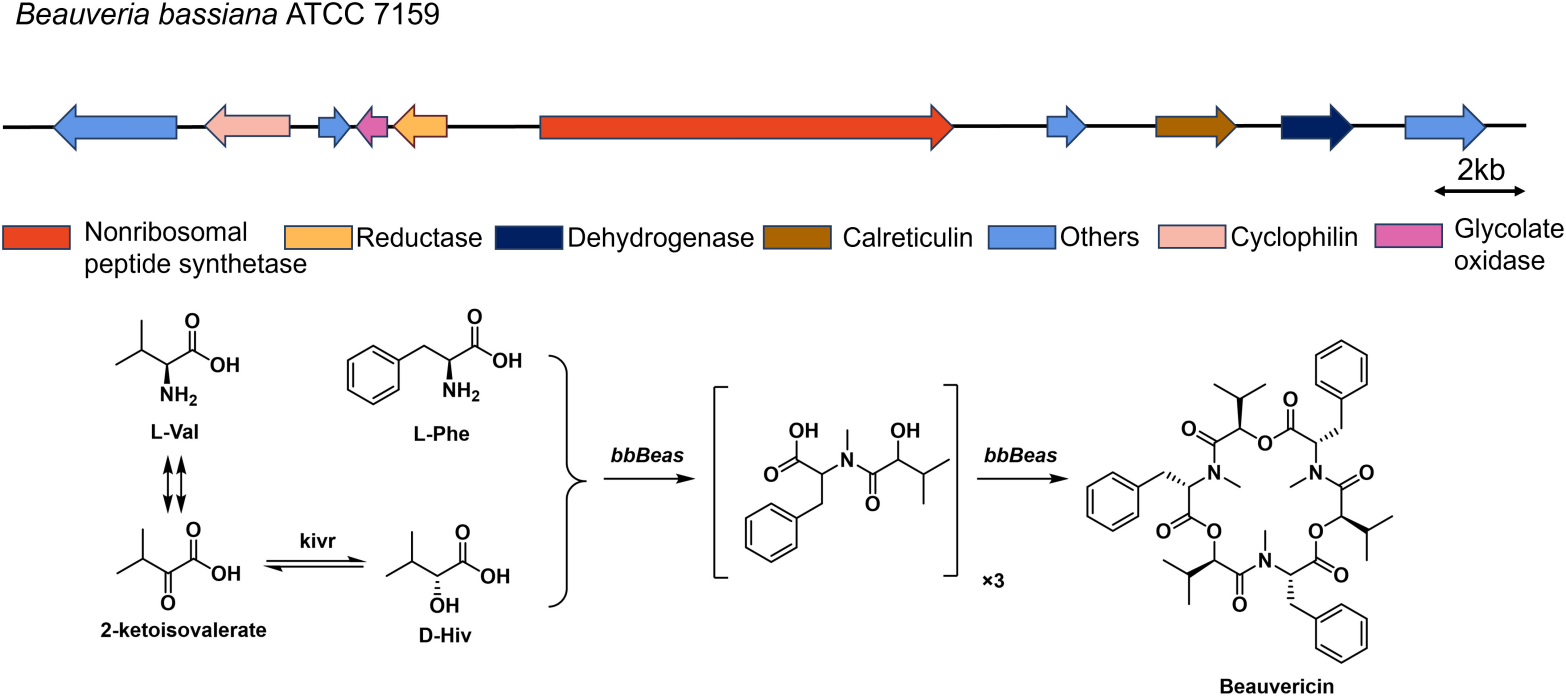
The biosynthetic gene cluster of beauvericin in *Beauveria bassiana* ATCC 7159 and the biosynthetic pathway of beauvericin.

Non-ribosomal peptides (NRPs) constitute a significant class of natural products and have a wide range of biological activities, including insecticidal, antifungal, antibacterial, and antitumor properties. Fungal sources are particularly prolific, among them, cyclooligomer depsipeptides (CODs) such as beauvericins, enniatins, bassianolides, and PF1022A, are cyclic trimers/tetramers synthesized from dipeptidol monomers (Yu et al., 2013). It is a cyclodepsipeptide composed of three dimers of phenylalanine and D-hydroxyisovaleric acid (D-Hiv). Its biosynthesis originates from valine-derived ketoisovalerate, which is reduced to D-Hiv by a 2-ketoisovalerate reductase (*kivr*). The nonribosomal peptide synthetase *bbBeas* then incorporates D-Hiv and phenylalanine to form dipeptide intermediates, catalyzing their cyclization to yield beauvericin (Figure 1; Kim et al., 2016). Beauvericin was first isolated from the mycelium of *B. bassiana*, and exhibited notable pharmacological and agricultural activities, including antitumor, insecticidal, antibacterial, and antiviral effects (Xu et al., 2008). The demand for environmentally friendly pest control agents underscores the need for efficient, high-yield production platforms for beauvericin. These valuable properties have driven considerable demand for efficient methods to produce beauvericin at high yields.

Current beauvericin production in native fungal producers like *B. bassiana* and *Fusarium redolens* was ∼20 and ∼62.4 mg/L, respectively (Xu et al., 2008; Xu et al., 2010). Sophisticated process optimization, i.e., kinetic model together with the stoichiometric relationships for biomass, substrate and product, leading to 299 mg/L beauvericin production from *F. redolens Dzf2* (Xu et al., 2011). Although the production of beauvericin in *Fusarium* sp. is slightly higher than that of *B. bassiana*, the use of *Fusarium* species is limited by their pathogenicity. Many *Fusarium* strains are known plant pathogens capable of causing *Fusarium* head blight (FHB) and producing harmful mycotoxins, such as fusaproliferin, fusaric acid, and moniliformin, alongside beauvericin and enniatins (Decleer et al., 2019). Heterologous expression in conventional hosts like *Escherichia coli* has also been attempted but resulted in low yields (∼8 mg/L), even with precursor supplementation (Xu et al., 2008). Co-expression of *bbBeas* and *kivr* the *Saccharomyces cerevisiae* BJ5464-NpgA led to 105.8 ± 2.1 mg/L beauvericin (Yu et al., 2013). However, these yields remain insufficient for large-scale applications, highlighting the need for more efficient heterologous production platforms.

To overcome these limitations, alternative microbial chassis have been explored. For instance, *Lactococcus lactis* has been used for the safe and cost-effective production of antimicrobial peptides (Tanhaieian et al., 2018). *Bacillus subtilis*, capable of secreting peptides and naturally producing compounds via non-ribosomal peptide synthetases (NRPS) and polyketide synthases (PKS), has enabled the synthesis of enniatin (Zobel et al., 2015). Similarly, *Aspergillus oryzae*, which lacks active secondary metabolite pathways involving harmful byproducts, presents a promising host for heterologous peptide synthesis (Yoshimi et al., 2018). An ideal production platform should therefore combine safety, high NRPS expression capacity, and efficient extracellular secretion.

Further yield improvements can be achieved through genetic engineering and fermentation optimization. For example, codon optimization, increased gene copy number, and gene fusion enhanced epoxomicin production in *E. coli* (Messenger et al., 2024). In *A. oryzae*, the yield of the cyclic dipeptide cyclo-TA was increased through substrate feeding (Qi et al., 2022). Similarly, split-expression of beauvericin biosynthetic genes in yeast increased production by 47.45% (Yin et al., 2024).

While several fungal hosts have been explored for heterologous NRP production, each presents trade-offs between genetic accessibility, precursor supply, and biosynthetic capacity. An ideal chassis should combine high innate peptide output, genetic tractability, and safety for scalable fermentation. Here, we report the development of the filamentous fungus *Emericellopsis* sp. XJ1056 as a powerful chassis for high-yield beauvericin biosynthesis. *Emericellopsis* sp. XJ1056, with its native high-titer antiamoebin production and absence of pathogenic traits, represents an underexplored yet promising candidate for platform development. We established a robust genetic toolkit for this strain, including a Δ*ku70* mutant to enhance homologous recombination efficiency. By deleting the genes *antD* and *helA* that are responsible for the synthesis of the main products antiamoebins and helvolic acid, we obtained an almost byproduct-free chassis, making it convenient for the separation of the target compounds (Supplementary Figure 2-2). Subsequently, we achieved *de novo* beauvericin synthesis by integrating the *bbBeas* synthetase gene and the precursor supply gene (*kivr*). By optimizing the fermentation conditions and codon usage, the yield of beauvericin reached 921.24 mg/kg. This study not only established a high-yield biosynthesis for beauvericin, but also demonstrated *Emericellopsis* sp. XJ1056 as a highly promising platform for the engineered production of other valuable non-ribosomal peptides.

## Materials and methods

### Strains and culture conditions

*Emericellopsis* sp. XJ1056 was originally isolated from the Kanas Lake, Xinjiang Province, China, in July 2018. The strain was cultured on YM solid medium (malt extract 10 g/L, yeast extract 2 g/L, agar 20 g/L) and incubated at 28°C for 10 days. Spores were harvested using 0.1% Triton X-100 solution. For solid medium cultures, each plate was inoculated with 1 × 10^5^ spores and incubated at 28°C for 10 days. Detailed medium compositions are provided in the Supporting Information. For liquid cultures, the same YM medium without agar was used, inoculated with 1 × 10^7^ spores in 100 mL medium within 250 mL flasks, and incubated at 28°C with shaking at 220 rpm for 7 days. Where indicated, cultures were supplemented with D-hydroxyisovaleric acid (D-Hiv) and phenylalanine each at a final concentration of 15 mM. Other strains used in this study are provided in Supplementary Table 1.

### DNA manipulation and plasmid construction

Fungal genomic DNA was extracted as previous reports (Chen et al., 2013). PCR amplifications were performed using 2× Phanta Max Master Mix (Vazyme Biotech, Nanjing, China). DNA fragments were assembled using the ClonExpress MultiS one-step cloning kit (Vazyme Biotech, Nanjing, China). PCR products were purified with the Axygen^®^ DNA Gel Extraction Kit (Corning, NY, United States). Plasmid preparation was carried out with the TIANprep Rapid Mini Plasmid Kit (Tiangen Biotech, Beijing, China). *E. oli* NEB 10-Beta (New England Biolabs, Ipswich, MA) was used for plasmid propagation. Superstain DNA marker (Cowinbioscience, Jiangsu, China) was used for electrophoresis.

The native *bbBeas* gene (Sequence ID: EU886196.1) was amplified from *B. bassiana* ATCC 7159. Its codon usage was optimized for *Emericellopsis* sp. XJ1056 using OPTIMWIZ, and the optimized sequence (*opbbBeas*) was chemically synthesized by GENEWIZ (Suzhou, China). Detailed codon-optimization data are provided in the Supporting Information.

### Genetic transformation of *Emericellopsis* sp. XJ1056

Four genetic transformation methods include polyethylene glycol-mediated protoplast transformation (PMT), endonuclease-mediated protoplast transformation (REMI), electroshock transformation and *Agrobacterium tumefaciens*-mediated transformation (ATMT) were attempted for the genetic transformation of *Emericellopsis* sp. XJ1056. Detailed protocols for REMI, electroporation and ATMT followed established reports. The PMT method was adapted from a published protocol with minor modifications (Wang et al., 2022). Briefly, *Emericellopsis* sp. XJ1056 spores from a 10-day culture on YM agar were inoculated into YM liquid medium and grown for 11 h at 28°C with shaking at 220 rpm. Mycelia were collected and treated with an enzyme mixture containing 5 mg/mL lysing enzymes (Sigma-Aldrich) and 2 mg/mL yatalase (Takara Bio) in osmotic buffer (1.2 M MgSO_4_⋅7H_2_O, 10 mM PBS, pH 5.8) for 2.5 h at 37°C with gentle shaking. Protoplasts were purified by centrifugation in trapping buffer (0.6 M sorbitol, 0.1 M Tris–HCl, pH 7.0) and washed with STC buffer (1.2 M sorbitol, 10 mM CaCl_2_, 0.1 M Tris–HCl, pH 7.5). For transformation, 5–15 μg of linear DNA was mixed with 100 μL protoplasts and incubated on ice for 50 min, followed by addition of 600 μL PEG solution (60% PEG 6000, 50 mM CaCl_2_, 50 mM Tris–HCl, pH 7.5). After 20 min at room temperature, the mixture was plated on CD-sorbitol medium with appropriate antibiotics. Transformants were selected after 5–7 days at 28°C and verified by PCR.

To improve homologous-recombination efficiency, the *ku70* gene was disrupted in the wild-type strain using a deletion cassette (uku70-PtrpC-hygB-dku70), yielding the Δ*ku70* mutant. Subsequent engineered strains were constructed by introducing target gene fragments into the Δ*ku70* background via PMT.

### Quantitative analysis of antiamoebins and beauvericin

Culture were dried at 65°C for 24 h prior to extraction. Metabolites were extracted three times with ethyl acetate. The combined extracts were concentrated under reduced pressure and redissolved in 5 mL methanol. Analysis was performed on an Agilent 1290 II UHPLC system coupled to an Agilent G6125B single quadrupole mass spectrometer (Agilent Technologies, United States). Separation used a ZORBAX Eclipse Plus C18 column (2.1 × 100 mm, 3.5 μm) at 28°C with a gradient of solvent A (H_2_O + 0.1% formic acid) and solvent B (acetonitrile + 0.1% formic acid) at a flow rate of 0.35 mL/min: 0–12 min, 5–95% B; 12–15 min, 95% B; 15–18 min, 5% B. Detection was by UV absorbance from 200 to 600 nm, with quantitation at 210 nm using calibration curves constructed with standard solutions of antiamoebins (125–2,000 μg/mL and beauvericin (62.5–500 μg/mL).

### Analysis of *bbBeas* and *opbbBeas* expression levels

The wild-type *Emericellopsis* sp. XJ1056 (WT) and the engineered strains Δ*ku70-bbBeas-kivr* and Δ*ku70-opbbBeas-kivr* were cultivated on YES agar media overlaid with sterile cellophane for 7 days at 28°C. Total RNA was extracted from the mycelia using TRIzol reagent (Invitrogen, Carlsbad, CA), and reverse transcription quantitative PCR (RT-qPCR) were conducted using PowerUp™ SYBR™ Green Master Mix (Thermo Fisher, Vilnius, Lithuania) on an Applied Biosystems™ 7500 Real-Time PCR System (Thermo Fisher). The RT-qPCR was performed to assess the transcript levels of *antD*, *bbBeas*, and *opbbBeas*. The translation elongation factor (TEF) gene was used as an endogenous reference for normalization. Expression levels were calculated using the comparative ΔΔCt method, with relative quantification defined as 2^–ΔΔCt^. Each experiment included three independent biological replicates.

### Statistical analysis

Statistical analyses were conducted using Prism GraphPad software (version 8.0). Data were presented as mean ± SD. Comparisons were performed by one-way analysis of variance (ANOVA) followed by Tukey’s HSD test. A *p*-value < 0.05 was considered statistically significant.

## Results

### Screening and identification of a high-yield polypeptide-producing fungus, *Emericellopsis* sp. XJ1056

**FIGURE 2:**
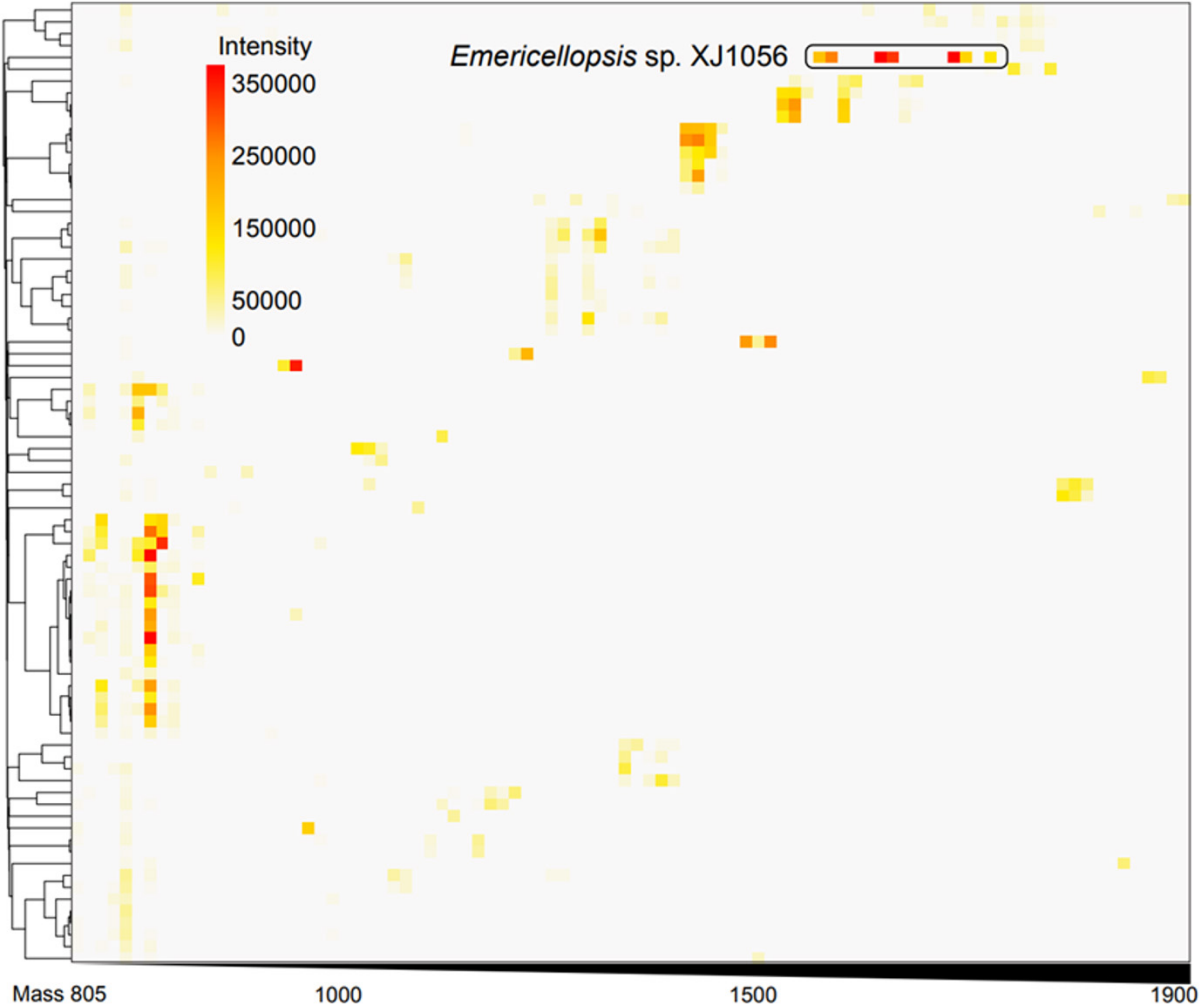
Heat map and hierarchical clustering of the MALDI-TOF MS spectra of secondary metabolites extracts from 164 fungal isolates. The abscissa represents the molecular weight of the compound. The ordinate represents different strains. The gradient color indicates the intensity of each ion signal.

**FIGURE 3:**
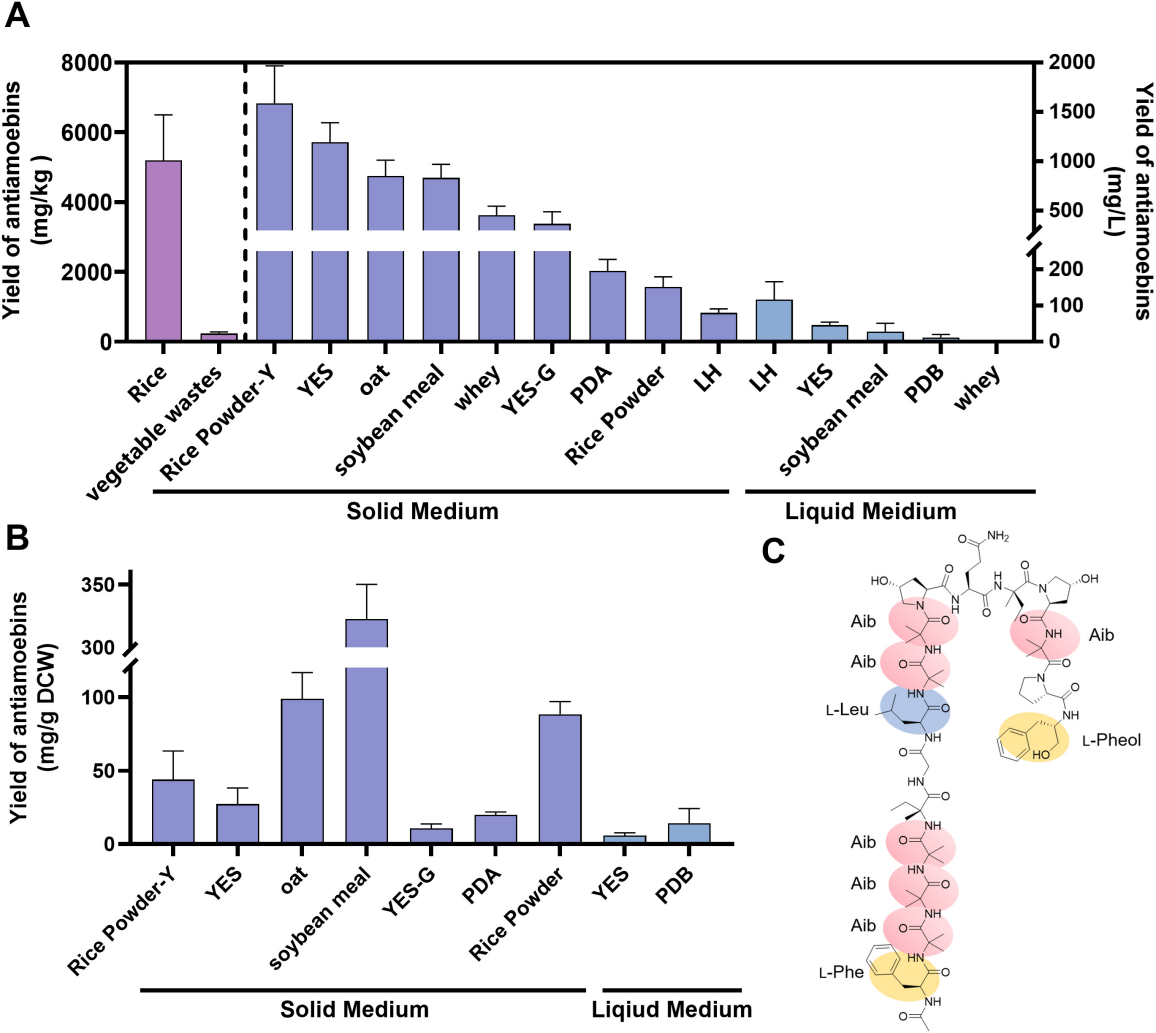
Yield of antiamoebins in *Emericellopsis* sp. XJ1056 fermented on different media. **(A)** The unit of titers was calculated in mg/L (right *y*-axis) or mg/kg (left *y*-axis). **(B)** The unit of titers was calculated in mg/g DCW. Rice Powder-Y, Rice powder medium added with yeast extract; YES-G, replacement of sucrose with glucose in YES; LH, lignocellulosic hydrolysate. **(C)** The chemical structure of antiamoebin I. Phe, phenylalanine; Aib, α-aminoisobutyric acid; Leu, leucine; Pheol, phenylalaninol.

To identify a suitable host for high-yield beauvericin production, we first screened 164 fungal strains isolated from Xinjiang soils using MALDI-TOF mass spectrometry. Among these, 125 strains exhibited signals within the peptide-relevant molecular weight range (800–2,000 Da) (Figure 2). Six isolates showing the strongest signals were selected for solid-state fermentation on rice medium. Ethyl acetate extracts of cultured metabolites were analyzed by LC-MS. Through the further isolation and purification, we confirmed that isolate XJ1056 produces antiamoebins at up to 5192.63 mg/kg dry weight, which was the highest production of peptide among the six strains, along with helvolic acid as a major byproduct (3 g/kg) (Song et al., 2023). Because antiamoebins and beauvericin share biosynthetic precursors derived from pyruvate (Figure 3C) (i.e., phenylalanine, α-aminoisobutyric acid, and leucine in antiamoebins; D-Hiv and phenylalanine in beauvericin), isolate XJ1056 was selected for subsequent engineering efforts.

Genome sequencing of strain XJ1056 was performed as previously reported (Song et al., 2023), and all predicted proteins were compared against the NR database. Among them, 7042 proteins (82.26%) showed highest similarity to those of *Emericellopsis* species, confirming the strain as *Emericellopsis* sp. XJ1056.

### Optimization of fermentation medium for antiamoebin production

To evaluate the peptide-production capacity of *Emericellopsis* sp. XJ1056, we tested multiple solid and liquid media for antiamoebin synthesis. Among solid media, YES medium yielded 1191.78 mg/L, significantly higher than PDA (195.85 mg/L) (Figure 3A). Due to that one of the major differences between these two commonly used media was carbon source, replacement of sucrose in YES with glucose (YES-G) was performed, which reduced production to 368.04 mg/L, indicating a preference for sucrose (Figure 3A). Soybean meal solid medium, commonly used in industrial fermentations, yielded 832.52 mg/L, but showed the highest unit production (322.61 mg/g DCW), which was 10.85-fold higher than that on YES medium (Figure 3B). Notably, although the total antiamoebin titer on YES solid medium was the highest among the common synthetic media tested, its yield per unit biomass (mg/g DCW) was relatively low (Figure 3B). This likely resulted from the substantially higher biomass accumulation on YES (46.43 g/L) compared to that on PDA (9.81 g/L), suggesting that YES medium promotes vigorous fungal growth but does not proportionally enhance the specific production of antiamoebins.

Rice or oat solid media used for fermentation of *Epicoccum nigrum* (Mapari et al., 2008) and *Penicillium simplicissimum* (Shah et al., 2022) were also tested. Among these cereal-based media, oat medium performed comparably to soybean meal, whereas rice medium achieved the highest total yield of 5192.63 mg/kg, substantially exceeding previously reported values (Figure 3A). In contrast, liquid cultures produced markedly lower titers: 45.93 mg/L in YES, 27.85 mg/L in soybean meal broth, and 12.12 mg/L in PDB (Figure 3A), indicating that solid-state fermentation is more conducive to high-level polypeptide production by this strain.

To enable the greener production of beauvericin by *Emericellopsis* sp. XJ1056, the use of low-cost agricultural wastes as substrates were tested, including vegetable wastes, whey, rice powder and lignocellulosic hydrolysate (LH). Whey can be used as a fermentation medium for the production of lipids, organic acids and alcohols, and can be used as a substitute for lactose to reduce fermentation costs (Carranza-Saavedra et al., 2023). Lignocellulosic hydrolysate (LH) derived from agricultural wastes or energy crops can be used as cheap hydrophobic carbon sources for fermentation (Wei et al., 2021). In the large-scale production of vegetables and fruits, 10–60% of the products ultimately become agricultural waste (Liu, 2023). *Emericellopsis* sp. XJ1056 successfully utilized vegetable waste, whey, rice powder, and lignocellulosic hydrolysate (LH) for antiamoebin synthesis with yield ranged from 80.25 to 454.06 mg/L, supporting the potential for low-cost industrial production (Figure 3A).

### Development of the genetic transformation system

**FIGURE 4:**
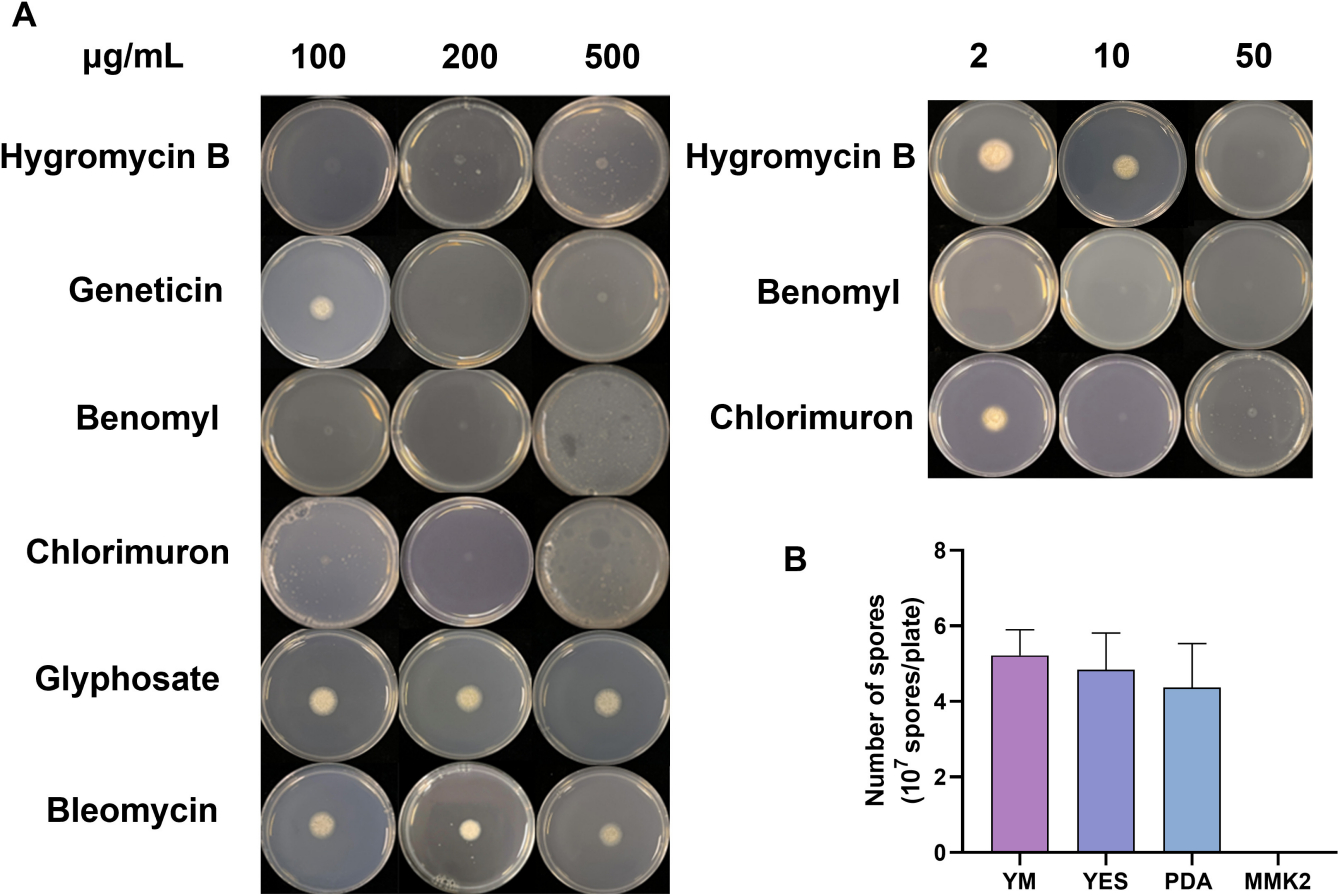
Development of the genetic transformation system. **(A)** Resistance tests of *Emericellopsis* sp. XJ1056 on different concentrations of antibiotics. **(B)** The number of spores on different culture media.

To enable genetic manipulation of *Emericellopsis* sp. XJ1056, we first determined its antibiotic sensitivity. The wild-type strain was resistant to glyphosate and bleomycin but sensitive to hygromycin (50 μg/mL), geneticin (200 μg/mL), benomyl (2 μg/mL), and chlorsulfuron (10 μg/mL) (Figure 4A). Among various sporulation media tested, YM agar yielded the highest spore count (5.22 × 10^7^ spores/plate) (Figure 4B and Supplementary Figure 1-1). Of four transformation methods evaluated, i.e., polyethylene glycol-mediated protoplast transformation (PMT), restriction enzyme-mediated integration (REMI), electroporation, and *Agrobacterium tumefaciens*-mediated transformation (ATMT), only PMT generated positive transformants. The failure of *Agrobacterium tumefaciens*-mediated transformation (ATMT) was likely because *Emericellopsis* sp. XJ1056 is not a suitable host for *A. tumefaciens* infection under the conditions tested. For restriction enzyme-mediated integration (REMI), the choice of restriction enzyme is critical; the specific enzyme used may not have created compatible integration sites in the genome. Finally, for electroporation, the applied electric shock conditions, while standard, may have been suboptimal for this strain, leading to high protoplast mortality and preventing the recovery of stable transformants.

**FIGURE 5:**
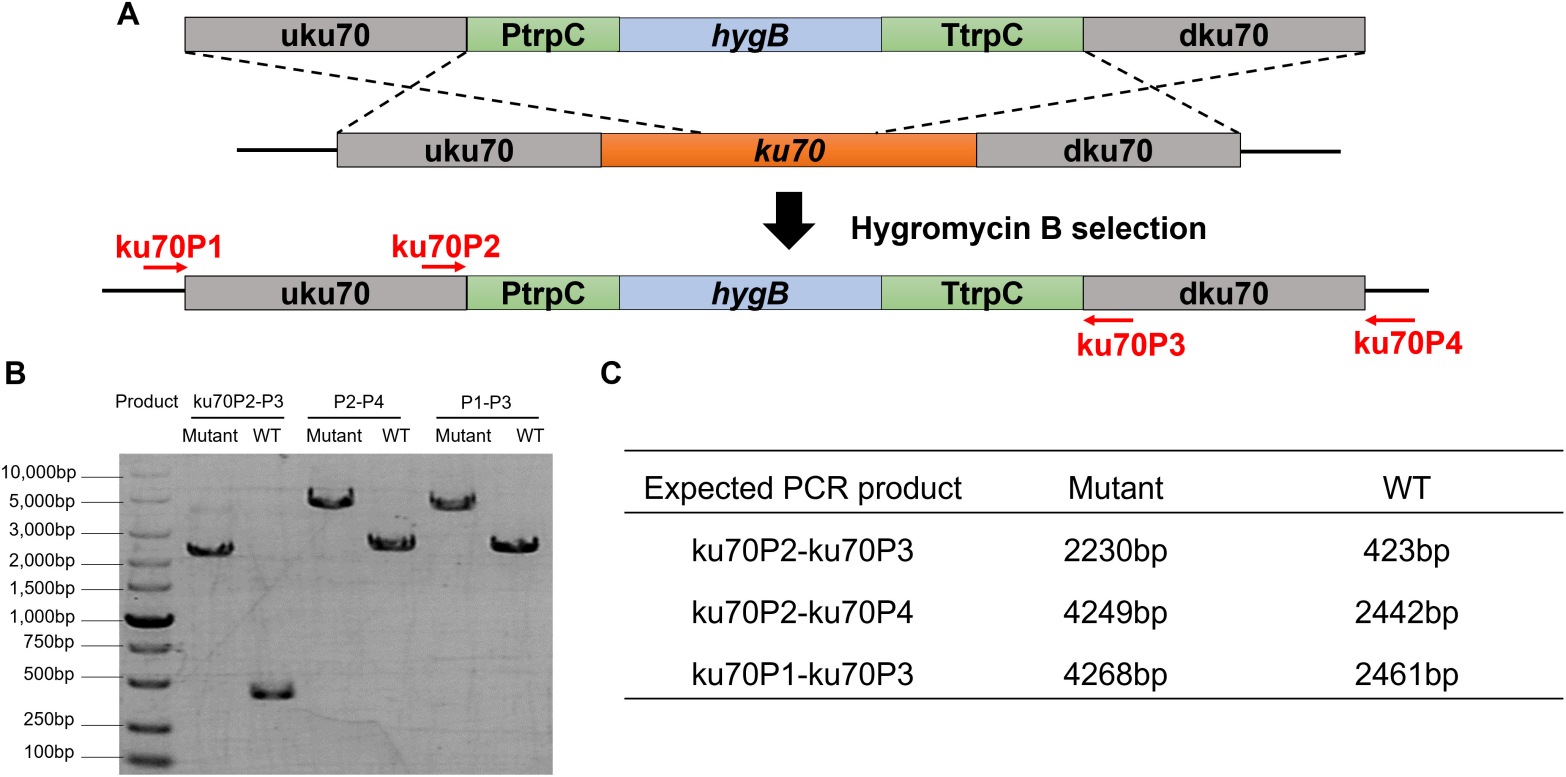
Construction and transformation of *ku70* deletion strain. **(A)** Schematic graph of *ku70* deletion. **(B)** PCR products for representative isolates of the wild type XJ1056 strain (WT, control) and the Δ*ku70* mutant. **(C)** The expected lengths of the PCR products amplified by the appropriate primers using the wild type or the mutant *Emericellopsis* sp. XJ1056 genomic DNA as the template.

To improve homologous recombination efficiency, we disrupted the *ku70* gene. The ku70/ku80 protein complex is related to non-homologous end-joining (NHEJ) pathway. By deleting *ku70* gene, non-homologous recombination can be diminished or prevented (Carvalho et al., 2010). Using a *ku70* deletion cassette (uku70-PtrpC-hygB-dku70), we obtained three correct Δ*ku70* mutants among 32 transformants (9.4% successful rate), as verified by PCR (Figure 5). The mutation was stably inherited over three generations. Further deletion mutation in following sections showed that the homologous recombination efficiency was improved up to 50–73.68% after the *ku70* gene knockout, and the integration locus markedly influenced transformation success, whereas fragment length and number had minimal impact (Supplementary Table 3). Comparing the morphology and product profiles showed that the *ku70* deletion does not alter the key physiological traits of the host, and the Δ*ku70* strain can serve as a starting chassis cell for further engineering.

### *De novo* biosynthesis of beauvericin

**FIGURE 6:**
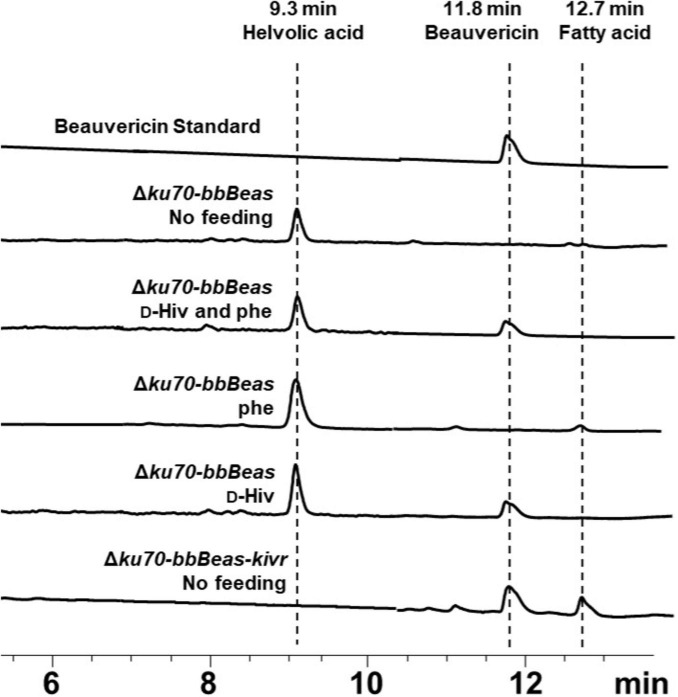
Beauvericin production under different precursor-feeding conditions on rice medium for 10 days (UV detection wavelength was 210 nm). The strain Δ*ku70-bbBeas-kivr* (*bbBeas* and *kivr* insertion) can produce beauvericin without precursor supplementation, maeanwhile the helvolic acid disappeared due to the *kivr* insertion.

First the whole 16-kb fragment with gene *bbBeas*, marker and flanking regions was attempted to be introduced into the Δ*ku70* strain by PMT after the promoter of *antD*, the gene encoding the NRPS that synthesizes antiameobins (Song et al., 2024). However, no transformant was successfully detected, likely due to the large size of foreign DNA fragment. Previous study showed that multiple homologous recombination could efficiently integrated large foreign DNA fragments in one transformation event (Chiang et al., 2021). We then employed this strategy using three overlapping fragments, each flanked by 1-kb homology arms, to successfully assemble the entire *bbBeas* sequence after the promoter of *antD* in the Δ*ku70* strain, yielding Δ*ku70-bbBeas*, which was verified by PCR with 4 pairs of primers (Supplementary Figure 3). Beauvericin production was then evaluated on rice medium under four precursor-feeding conditions: no feeding, feeding both D-Hiv and phenylalanine, feeding phenylalanine alone, and feeding D-Hiv alone (Figure 6). Production was detected only in cultures supplemented with D-Hiv (with or without phenylalanine), indicating that endogenous phenylalanine was sufficient but D-Hiv availability was limiting.

**FIGURE 7:**
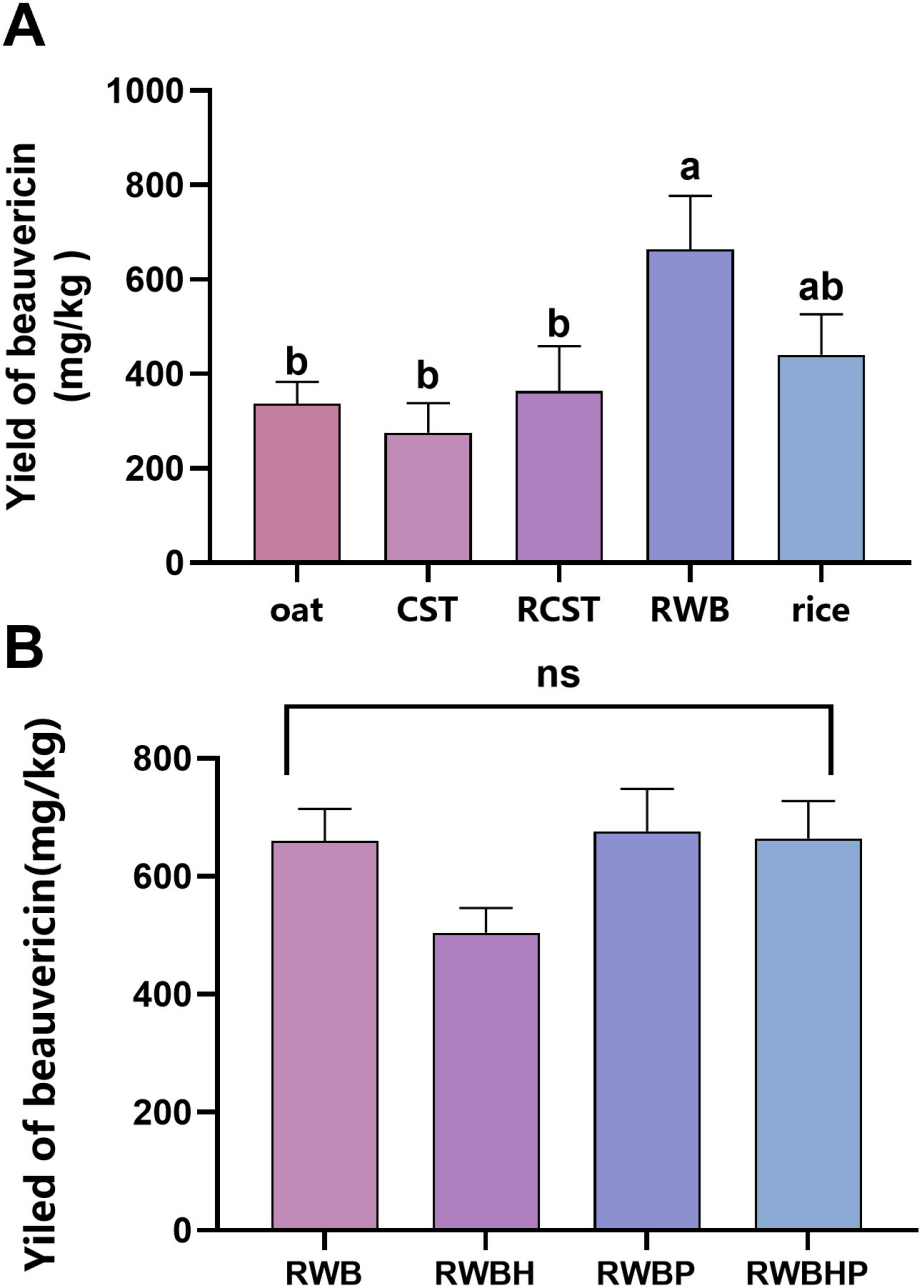
Production of beauvericin in the engineered strain under different conditions. **(A)** Yield of beauvericin on various optimized solid media. CST, corn starch solid medium; RCST, rice supplemented with corn starch; RWB, rice supplemented with wheat bran. Data are mean ± SD (*n* = 3). Different lowercase letters above bars indicate statistically significant differences among treatments according to Tukey’s HSD test (*p* < 0.05). **(B)** Effect of precursor supplementation on beauvericin production in strain Δ*ku70-bbBeas-kivr* cultivated on RWB medium. RWB, rice with wheat bran; RWBH, RWB plus D-Hiv; RWBP, RWB plus phenylalanine; RWBHP, RWB plus both D-Hiv and phenylalanine.

To enable *de novo* synthesis, the *kivr* gene encoding a 2-ketoisovalerate reductase from *B. bassiana* ATCC 7159 was introduced after the promotor of *helA* gene, which generated Δ*ku70-bbBeas-kivr* (Supplementary Figure 4). The *kivr* catalyzes the formation of D-Hiv from ketoisovaleric acid that is derived from valine (Yu et al., 2013). The *helA* gene encoding terpene cyclase is involved in the biosynthesis of another major product of *Emericellopsis* sp. XJ1056, helvolic acid (Lv et al., 2017), which ensures the efficiency of its promoter. In addition, disruption of *helA* gene diminished the production of helvolic acid and consequently simplify the product purification (Figure 6). As a result, the strain Δ*ku70-bbBeas-kivr* produced beauvericin without precursor supplementation with the yield of 440.10 mg/kg when cultured on rice solid medium for 10 days (Figure 7A).

### Fermentation optimization and precursor feeding for beauvericin production

Using the rice-based solid medium that supported the highest antiamoebin yield, strain Δ*ku70-bbBeas-kivr* produced 440.10 mg/kg beauvericin, which was decreased by 92% compared to the antiamoebin titer, showing the need for optimization to further enhance the yield. Supplementation of the rice medium with wheat bran (RWB) significantly increased beauvericin production to 663.40 mg/kg, whereas additions such as corn starch (CST/RCST) were less effective (Figure 7A).

Previous results showed that the addition of yeast extract into the rice powder medium significantly increased the yield of antiamoebins as well as the biomass of *Emericellopsis* sp. XJ1056. However, the yield per unit biomass was lower. This is likely attributable to the higher biomass accumulation (36.69 g/L with yeast extract vs. 1.73 g/L on rice powder alone), which suggests that yeast extract promotes growth more strongly than it enhances specific peptide production. To achieve an optimal balance between biomass and product synthesis for beauvericin, we adjusted the ratio of rice to yeast extract in the medium. Strains were cultured on media with rice-to-yeast extract ratios of 3:1, 15:1, 30:1, and 50:1. Contrary to our expectation, excessive yeast extract reduced beauvericin yield, and none of the adjusted ratios improved production compared to the baseline (Supplementary Figure 8). Consequently, rice supplemented with wheat bran (RWB), which gave the highest yield without requiring yeast extract optimization, was selected for all subsequent fermentations.

Another possible reason for the much less yield of beauvericin may be the depletion of precursor(s), i.e., phenylalanine and/or D-Hiv. However, substrate feeding experiments showed that adding D-Hiv, phenylalanine, or both to RWB medium did not significantly enhance beauvericin production in Δ*ku70-bbBeas-kivr*, suggesting that precursor supply was not the limiting factor (Figure 7B).

### Engineering of the beauvericin synthetase *bbBeas* and its gene

**FIGURE 8:**
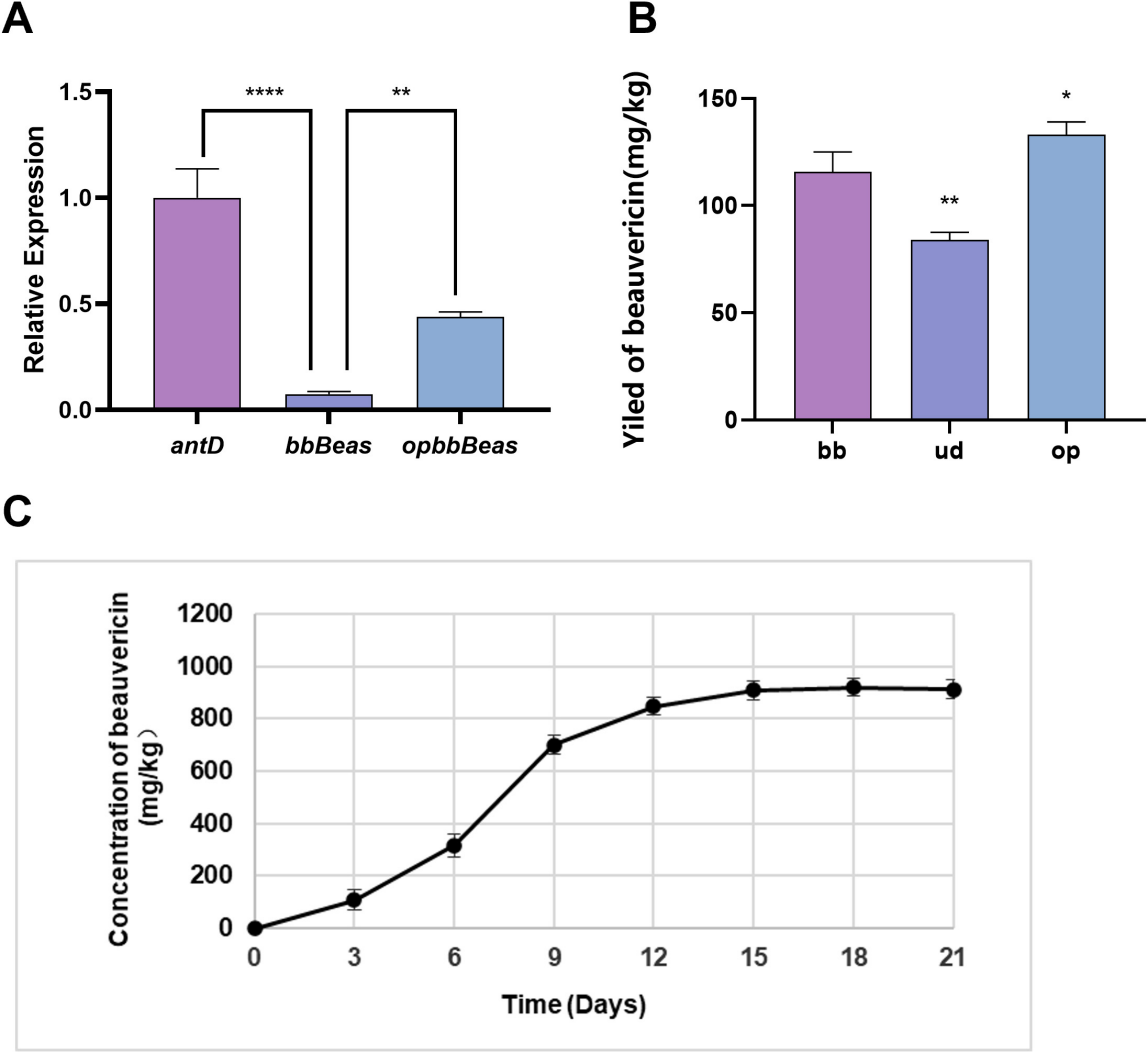
Enhancement of beauvericin production through codon optimization and time course profiling. **(A)** Relative transcript levels of *antD* (wild-type control), *bbBeas*, and *opbbBeas* determined by RT-qPCR. ****P < 0.0001 vs. *antD*; **P < 0.01 vs. *bbBeas* (Tukey’s HSD test). **(B)** Beauvericin yields of engineered strains cultivated for 10days on rice supplemented with wheat bran medium, D-Hiv, and phenylalanine. Strains: Δ*ku70-bbBeas* (bb), Δ*ku70-udbbBeas* (ud, split-expressed variant), Δ*ku70-opbbBeas* (op, codon-optimized). Data are mean ± SD (*n* = 3). **P < 0.01, *P < 0.05 vs. bb group (Tukey’s HSD test). **(C)** Time-course production of beauvericin by strain Δ*ku70-opbbBeas-kivr* grown on rice supplemented with wheat bran medium. The highest titer (921.24mg/kg) was achieved at 18days.

To further enhance the yield of beauvericin, we focused on engineering the key enzyme, beauvericin synthetase *bbBeas*. RT-qPCR analysis revealed that the transcript level of the heterologous *bbBeas* gene in strain Δ*ku70-bbBeas-kivr* was only about 7% of that of the native *antD* gene in the wild-type strain (Figure 8A). We hypothesized that this low expression might stem from suboptimal translation efficiency or mRNA instability due to codon mismatch. To address this, we redesigned the *bbBeas* coding sequence according to the codon usage preference of *Emericellopsis* sp. XJ1056, yielding an optimized version designated *opbbBeas*. This gene was inserted downstream of the strong *antD* promoter using the same strategy as the *bbBeas* gene, generating strain Δ*ku70-opbbBeas* (Supplementary Figure 7). When cultured for 10 days on rice medium supplemented with wheat bran and both precursors (D-Hiv and phenylalanine), the codon optimized strain produced 133 mg/kg beauvericin, representing a 15% increase over the non-optimized control (Figure 8B). The *kivr* gene was then introduced under the control of the *helA* promoter into this background, creating the final engineered strain Δ*ku70-opbbBeas-kivr*. RT-qPCR confirmed that the transcript abundance of *opbbBeas* in this strain was 5.29-fold higher than that of the original *bbBeas* (Figure 8A). Time course fermentation on rice supplemented with wheat bran medium for 21 days showed that beauvericin accumulation peaked at 921.24 mg/kg after 18 days of cultivation (Figure 8C).

In parallel, we evaluated a split-expression strategy that had been reported to enhance NRPS expression in *S. cerevisiae* by dissecting the synthetase into two catalytically independent subunits with artificially reconstructed thiolation domains. In *S. cerevisiae* BJ5464-NpgA, split-expression of the beauvericin synthetase gene *bbBeas* achieved 47.45% enhancement of titer when the downstream fragment initiated at residue R1009 within the α5 helix (Yin et al., 2024). We applied this split-expression approach to *bbBeas* in *Emericellopsis* sp. XJ1056. The upstream and downstream segments of *bbBeas* were placed under the control of the *antD* and *helA* promoters, respectively, resulting in the engineered strain Δ*ku70-udbbBeas* (Supplementary Figures 5, 6). To our surprise, after 10 days of fermentation, split-expression of *bbBeas* led to a noticeable decrease in beauvericin yield (Figure 8B).

## Discussion

In this study, we established *Emericellopsis* sp. XJ1056 as a highly efficient platform for the *de novo* biosynthesis of beauvericin. The selection of this filamentous fungus was guided by its native capacity to produce high titers of antiamoebins (up to 5.2 g/kg), reflecting its inherent proficiency in nonribosomal peptide assembly. By developing a robust genetic toolkit, including a Δ*ku70* mutant to improve homologous recombination efficiency, and integrating the heterologous *bbBeas* gene together with the precursor-supplying *kivr* gene under strong endogenous promoters, we engineered a strain capable of synthesizing beauvericin without external precursor feeding. Through systematic optimization of fermentation conditions and codon usage, a final yield of 921.24 mg/kg was achieved.

The high beauvericin titer obtained here underscores the potential of *Emericellopsis* sp. XJ1056 as a chassis for complex natural product biosynthesis. Unlike conventional hosts such as *S. cerevisiae* or *E. coli*, this fungus possesses a naturally optimized metabolic background for peptide synthesis, reducing the need for extensive pathway engineering to supply precursors such as D-Hiv and phenylalanine. Notably, the failure of split-expression strategies to improve yields in contrast to their success in yeast suggests that the folding, modification, or interaction of nonribosomal peptide synthetases may be host-specific. This observation highlights the importance of tailoring expression strategies to the unique cellular environment of the chosen host.

Furthermore, the significant production boost achieved through codon optimization indicates that translational efficiency, rather than transcriptional capacity, was one of the major limiting factor in this system. The fact that precursor feeding did not further enhance yields supports the conclusion that metabolic flux was not the primary bottleneck; instead, post-transcriptional regulation and enzyme activity played decisive roles.

This work not only provides an efficient and sustainable route for beauvericin production but also establishes a generalizable platform for the biosynthesis of other high-value nonribosomal peptides. Future efforts could focus on dynamic regulation of key pathway genes and the application of similar engineering strategies to other complex peptide families. Moreover, the successful use of agricultural waste as a low-cost growth medium further improves the economic and environmental feasibility of scaling up production.

## Data Availability

The original contributions presented in this study are included in the article/Supplementary material, further inquiries can be directed to the corresponding authors.
