# Supplementary material for: Developing Emericellopsis sp. XJ1056 as a versatile fungal platform for high-yield biosynthesis of nonribosomal peptides: a case study on beauvericin
Source: Front Microbiol. 2026 Apr 16;17:1805553. doi: 10.3389/fmicb.2026.1805553 (PMC13128545; doi:10.3389/fmicb.2026.1805553)
Supplement: Supplementary file 1 [file Supplementary_file_1.docx]

**SUPPLEMENTARY INFORMATION FOR**

**Developing *Emericellopsis* sp. XJ1056 as a Versatile Fungal Platform for High-Yield Biosynthesis of Nonribosomal Peptides: A Case Study on Beauvericin**

**Table of Contents**

[Culture medium formula 3](#_Toc224673964)

[Optimization of beauvericin synthase *bbBeas* 4](#_Toc224673965)

[The methods of optimization 4](#_Toc224673966)

[The optimized sequence of *bbBeas* 5](#_Toc224673967)

[Other Genetic transformation methods of *Emericellopsis* sp. XJ1056 12](#_Toc224673968)

[Supplementary Figures and Tables 12](#_Toc224673969)

[Figure S1-1 The morphology of the *Emericellopsis* sp. XJ1056 wild type cultured on different media for 10 days. 12](#_Toc224673970)

[Figure S1-2 No-antibiotic control of the *Emericellopsis* sp. XJ1056 wild type. 12](#_Toc224673971)

[Figure S1-3 The morphology of the *Emericellopsis* sp. XJ1056 wild type (WT) and the Δ*ku70* strain cultured on YES medium for 10 days. 13](#_Toc224673972)

[Figure S2-2 The secondary metabolites produced by the *Emericellopsis* sp. XJ1056 wild type (WT) and Δ*helA*Δ*antD* strain after being cultured on the rice medium for 10 days. The secondary metabolites were almost non-existent after knocking out *helA* and *antD*. 14](#_Toc224673973)

[Figure S3 Construction and transformation of *bbBeas* insertion strain 14](#_Toc224673974)

[Figure S4 Construction and transformation of *kivr* insertion strain 15](#_Toc224673975)

[Figure S5 Construction and transformation of the upstream unit of *bbBeas* insertion strain 16](#_Toc224673976)

[Figure S6 Construction and transformation of the downstream unit of *bbBeas* insertion strain 16](#_Toc224673977)

[Figure S7 Construction and transformation of optimized *bbBeas* insertion strain 17](#_Toc224673978)

[Figure S8 The yield of beauvericin of the strain Δ*ku70-bbBeas-kivr* on different ratios of rice to yeast extract 18](#_Toc224673979)

[Figure S9 The standard curve of antiamoebins and beauvericin. 18](#_Toc224673980)

[Figure S10 HRESIMS spectrum of beauvericin and crude extract of the strain Δ*ku70-opbbBeas-kivr* 18](#_Toc224673981)

[Table S1 Strains and plasmids used in this study. 18](#_Toc224673982)

[Table S2 Primers used in this study. 21](#_Toc224673983)

[Table S3 The homologous recombination efffciency 28](#_Toc224673984)

[References 30](#_Toc224673985)

# Culture medium formula

Rice solid medium (rice 10 g and H_2_O 15 mL in 100 mL flask), Cabbage (vegetable wastes 30 g and H_2_O 15 mL in 100 mL flask), Rice Powder-Y (rice powder 60 g/L, yeast extract 20 g/L, agar 12 g/L), YES (yeast extract 20 g/L,MgSO_4_·7H_2_O 0.5 g/L, sucrose 150 g/L, ZnSO_4_·7H_2_O 0.01 g/L, CuSO_4_·5H_2_O 0.005 g/L, agar 20 g/L), oat solid medium (oat powder 60 g/L, agar 12 g/L), soybean meal solid medium (soybean meal 10 g/L, corn starch 20 g/L, KH_2_PO_4_ 2 g/L, FeSO_4_ 0.005 g/L, MnSO_4_ 0.006 g/L, CaCl_2_ 0.3 g/L, KCl 1 g/L, agar 20 g/L), whey solid medium (lactose 90 g/L, milk powder 10 g/L, ZnSO_4_·7H_2_O 0.02 g/L, H_3_BO_3_ 0.011 g/L, MnCl_2_·4H_2_O 0.005 g/L, FeSO_4_·7H_2_O 0.0016 g/L, CoCl_2_·5H_2_O 0.0016 g/L, CuSO_4·_5H_2_O 0.0016 g/L, (NH_4_)_6_Mo_7_O_24_·4H_2_O 0.0011 g/L, NaNO_3_ 6 g/L, KCl 0.52 g/L, MgSO_4_·7H_2_O 0.52 g/L, KH_2_PO_4_ 30.4 g/L, agar 20 g/L), YES-G (glucose 150 g/L, yeast extract 20 g/L, MgSO_4_·7H_2_O 0.5 g/L, ZnSO_4_·7H_2_O 0.01 g/L, CuSO_4_·5H_2_O 0.005 g/L, agar 20 g/L), PDA (potato starch 4 g/L, dextrose 20 g/L, agar 15 g/L), rice powder solid medium (rice powder 60 g/L, agar 12 g/L), lignocellulosic hydrolysate solid medium (glucose 14.2 g/L, xylose 5 g/L, acetic acid 0.4 g/L, yeast extract 2.5 g/L, agar 20 g/L). If preparing the liquid culture medium, no agar was added.

YM solid medium (malt extract 10 g/L, yeast extract 2 g/L, agar 20 g/L), MMK2 (gluconate 40 g/L, yeast extract 5 g/L, Murashige&SKoog 4.3 g/L, agar 20 g/L).

CD solid medium (glucose 10 g/L, 20× Nitrate salts 50 mL/L, Trace elements 1 mL/L, 20 g/L agar), CD-Sorbitol solid medium (glucose 10 g/L, 20×Nitrate salts 50 mL/L, Trace elements 1 mL/L, Sorbitol 218.6 g/L, agar 20 g/L). 20× Nitrate salts (NaNO_3_ 120 g/L, KCl 10.4 g/L, MgSO_4_·7H_2_O 10.4 g/L, KH_2_PO_4_ 30.4 g/L),Trace elements (ZnSO_4_·7H_2_O 2.2 g/L, H_3_BO_3_ 1.1 g/L, MnCl_2_·4H_2_O 0.5 g/L, FeSO_4_·7H_2_O 0.16 g/L, CoCl_2_·5H_2_O 0.16 g/L, CuSO_4_·5H_2_O 0.16 g/L, (NH_4_)_6_Mo_7_O_24_·4H_2_O 0.11 g/L).

Rice supplemented with wheat bran (rice 9.5 g, wheat bran 0.5 g and H_2_O 15 mL in 100 mL flask), rice supplemented with corn starch (rice 7 g, corn starch 3 g and H_2_O 15 mL in 100 mL flask), corn starch solid medium (corn starch 10 g and H_2_O 15 mL in 100 mL flask).

# Optimization of beauvericin synthase *bbBeas*

## The methods of optimization

The gene sequence was optimized using OPTIMWIZ according to the codon usage preference of *Emericellopsis* sp. XJ1056 *CDS sequences*, and chemically synthesized under the GENEWIZ outsource service (Suzhou, China).

OPTIMWIZ, the GENEWIZ codon optimization tool, has the capability to optimize multiple critical parameters to stabilize DNA fragments and improve gene expression efficiency. These parameters include: codon usage bias, GC-content, mRNA secondary structure, custom desired patterns (for example, increasing the efficiency of gene expression by modifying the ShineDalgarno sequence or Kozak sequence), custom undesired patterns (for example, regulating the efficiency of gene expression by modifying the RNA instability motifs), repeat sequences (direct repeat, inverted repeat, and dyad repeat), restriction enzyme recognition sites (deletion or insertion). CAI can be even improved from 0.00 to 0.93 in *Emericellopsis* sp. XJ1056 with OPTIMWIZ.

Codon Frequency Table Used:

| TTT | 0.01 | TCT | 0.01 | TAT | 0.01 | TGT | 0.00 |
| --- | --- | --- | --- | --- | --- | --- | --- |
| TTC | 0.02 | TCC | 0.01 | TAC | 0.02 | TGC | 0.01 |
| TTA | 0.00 | TCA | 0.01 | TAA | 0.00 | TGA | 0.00 |
| TTG | 0.01 | TCG | 0.02 | TAG | 0.00 | TGG | 0.01 |
| CTT | 0.01 | CCT | 0.01 | CAT | 0.01 | CGT | 0.01 |
| CTC | 0.03 | CCC | 0.02 | CAC | 0.02 | CGC | 0.02 |
| CTA | 0.01 | CCA | 0.01 | CAA | 0.02 | CGA | 0.01 |
| CTG | 0.02 | CCG | 0.01 | CAG | 0.03 | CGG | 0.01 |
| ATT | 0.02 | ACT | 0.01 | AAT | 0.01 | AGT | 0.01 |
| ATC | 0.02 | ACC | 0.02 | AAC | 0.02 | AGC | 0.02 |
| ATA | 0.00 | ACA | 0.01 | AAA | 0.01 | AGA | 0.01 |
| ATG | 0.02 | ACG | 0.02 | AAG | 0.04 | AGG | 0.01 |
| GTT | 0.01 | GCT | 0.02 | GAT | 0.02 | GGT | 0.02 |
| GTC | 0.03 | GCC | 0.03 | GAC | 0.04 | GGC | 0.03 |
| GTA | 0.01 | GCA | 0.02 | GAA | 0.02 | GGA | 0.01 |
| GTG | 0.02 | GCG | 0.02 | GAG | 0.04 | GGG | 0.01 |

## The optimized sequence of *bbBeas*

ATGGAGCCCCTCAAGAACGTCAACACCGGTCAGCCCTGCAGCACCGTCCCCTTCCCCGTCAGCGACGAGACCGTCGAGCACCTCAACGGCCTCTACGAGGAGATCAACCGCCGCTTCGGCCTGGACCGCGATGCCATCGAGACCATCCTCCCCTGCACCCCCTTTCAGTACGACGTCCTCGACTGCGCCGCCAACGACGCCCGCCATGCCGTCGGCCACGCCATGTACGAGATCTCGCAGCATGTCCACGTGCAGCGCTTTATCGCCGCCTGGCGCGAGACCGTCCGACGAACCCCTGCCCTCCGAGCTTGCACCTTCACGAGCACCACCGGCGAGAGCTTTCAGCTCGTGCTCCGCGAGAGCTTCGTCCTCTCGCGCATCTACTGGAGCAGCTCGAGCAGCCTCCAAGCCGCCGTCCTGAAGGATGAGACCACCGCTGCCATCGCCGGTCCCCGCTGCAATCGCCTGGTCCTCCTCGAGGACCCCGACACCCGCAAGCAGCTCCTCATCTGGGTCTTCCACCTCGCCCTCGTCGACTCGACCGTCCAAGAGCCTATCCTCCGCCGCGTCCTGGCCGCCTACAAAAGCGAGGACGATCAACTGGATTCGCTCCCTCTGACGCCTGATAGCTCGGGCGGCAGCGACAGCGACAGCCCTAGCACCCTCAAGATGCCCCGCGCCTTCGACCAAGAGAAAGCCACCCAATTCTGGCAGCGACAACTCAGCGGCCTGGACGCTAGCGTCTTCCCTCCCCTCAGCAGCCATCTCACCACCCCCAAGGCCGACGCCAAGATCGAACACTATATCTCGTGGCCCGCTAGCGCCGCTCAACACCGCTGGAGCAGCACGACGGTCTGCCAAGCCGCCCTCGCTGTCCTCCTCTCGCGCTACAGCCATAGCAGCGAGGCCCTCTTCGGCGTCGTCACCGAGCAAGTCTGCATGTTCGAAGGTCAGCGCCTCCTCATCAACGGTCCCACCCGCAGCGTCGTGCCTTTCCGCGTGCACTGCGGCCCCGAGCAGAGCGTCACCGATCTCCTCAAGAGCATCGCTAGCGACAACCACGACATGCGACAGTTCGCCCACGTCGGCCTCTGCAACATCAGCCGCATCGGCGACGATCAGAGCGCCGCCTGCCGCTTTCAGACCGTCCTCAGCGTCTCGAATCGACGCAGCAGCGAGGACGCCGCTAGCGGTGAGGTCCTGCAGATCCTCCAAGAGAGCGAGGGCTTCGCTCCCTGCGCTGATCGAGCCCTCCTCCTGCGCTGTGAGACCTCGCGCCAAGGTGCCCTGCTCGTCGCCCGCTATGACCAAGGCGTCATTGAGCCCCCTCAGATGGCCCGCTTCCTCCGACAGCTCGGCTGGCTCATGGAGCAGCTGCAGAGCGCTGCTGACGACGCCCTGAGCGTCAAGCAGCTCGACATCGTCACGCGCGAGGATCGCGCCGAGATCGACAGCTGGAACAGCGACGCTCTCGAAGTCCAAGAGAGCCTGCTGCACAGCGCCTTCGTCAAACGCGCCGCCGAAAGCCCTAGCGATCCTGCCGTCCTCAGCTGGGACGGCGCCTGGACCTACAGCGAGCTCGACAACGTCAGCTCGCGCCTCGCTGCCCATATCCGCAGCCTCGACCTGAGCCACGAGCAGCTCATCGTCCCCGTCTACTTCGAGAAGAGCAAGTGGGTCGTCGCTAGCATCCTGGCCGTCCTCAAAGCCGGCCATGCCTTCACGCTCATCGACCCCAAGGATCCCCCCGCCCGCACCACGCGAATCGTGCAGCAGACGAGCGCCAAGGTCGCCCTCACGAGCAAGCTCCACCAAGACACCGTCCAAGCCATCATCGGCCGCTGCATCGTCGTCGATGACGATTTCGTGCAGAGCCTCGGCAGCGCTTCGCAGTGCCAAGAGAAGAGCGAGCTCACGGTCAAGCCCCACAACCTCGCCTACGCCATCTTCACGAGCGGCAGCACCGGCGACCCCAAGGGCATCATGATCGAGCACCAAGCCTTCGCCTCGTGTGTCGCCAAGTTCGGCCCCGCTCTCATTCCCCACAACGCCCGCGCCCTGCAGTTTGCTAGCCACGGCTTCGGCGCCTGCCTCCTCGAAATTCTCCCCACCCTCCTCCGCGGCGGCTGCGTCTGTATTCCTAGCGACCTCGACCGAATGCATAACATCCCCGACTTCATCCGCCGCTACAACGTCAACTGGATGATGGCCACCCCCTCGTACATGACCACGTTCAAGCCCGAAGACGTCCCTGGCCTGCAGACGCTCATCCTCGTCGGTGAACAGATGAGCGCTAGCGTGAACGCTACCTGGGCTAGCCGCCTCGGCCTCTTCGACGGCTACGGTCAGAGCGAGAGCTGCAGCATCTGCTTCATCGGCAAGATCTCGCCTGTCAGCGAGGCCAACAACATTGGTCGAGCCGTCGGCGCTCACTCGTGGATCGTCCATCCCGACGACCCCGATCGACTGGCCCCTGTGGGCGCCGTCGGTGAGCTCCTCATCGAGTCGCCTGGCATCGCCCGCGGCTACATCGCCGCTCCCGCCACCGACCGCAACCCCTTCCTCGAAACGGCTCCTGCCTGGTACGCCCCCCGACAGCCCCCCACCGGCGTCAAATTCTATCGAACCGGTGACCTCGCCCGCTACGCTGCTGACGGCACCGTCGTCTGCCTGGGCCGCATTGACAGCCAAGTCAAGATCCGCGGTCAGCGCGTCGAGATGGGTGCCGTCGAAACCCGCCTCCGACAGCAAGTCCCTAGCGACATCACCGTGGTGGCTGAAGCCGTCAAGCGCTCGGGCAGCTCGGGTAGCACGGTCATTACCGCCTTCCTCATCGACAGCTCGGACAAAAACAACAGCAGCGCCGCTAGCGCCAAGGACGCCCGCATCCTCGATCAGACCGCCACCCAAGAGATGAACGCCAAGCTCTGCCAAGTCCTCCCTCCCCACAGCGTCCCTAGCTGCTACATCTGCATGCACGCCCTCCCTCGAACGGCCACCGGCAAGGTCGACCGAAAGACGCTGCGAAGCATCGGCAGCAAGCTCCTCGAGCAGCAAGCCTACAAGAAGAGCCCCGAGACGATGCAAAAAAGCAAGAGCGCCGAGACCCTCGAGACGGGCCCCGAGGCCCGCCTGAAAGAGGTCTGGCTGCAGAGCTTCAACCTCGAGCCTGCTAGCCCCAAGTGCGGCGCTAGCTTCTTCGAGCTGGGCGGCGATTCGATCACCGCCATCAAGATGGTCAACATGGCCCGCGCTGCCGGCCTCGAGCTCAAGGTCAGCGACATCTTTCAGAACCCCACCCTCGCCCGCCTCCAAGCCGTCATGAGCGGCGACAGCACCCCTAGCACGATCACCACGCCCTTCGCCACCATCCCCGCCTCGACCTGGGACGGCCCTGTCGAGCAGAGCTACAGCCAAGGCCGCCTCTGGTTCCTCGACCAACTCGATATCGGCGCCGTCTGGTACCTCATTCCCTATGCCGTCCGCATGCGCGGCGCCCTCAACATCGATGCTCTCCGAGCTGCCCTCCTGGCTCTCGAGCAGCGCCACGAGACCCTCCGCACCACCTTCGAGAATCAGAACGGCGTCGGCGTGCAGATCGTGCATCAGCGCCTGGCCAAGGAGCTCAAGATCATCGACGCTAGCAGCCACGGTGATGATGGCTACCTGCAGCCCCTCGAGCAAGAGCAAACCACCCCCTTTGACCTCACCTGCGAGGCTGGCTGGCGCGCTAGCCTCATTTGCGTGGGCGAGGACCACCACGTCCTCAGCATCGTCATGCACCACATCGTCAGCGATGGCTGGAGCATCGATGTCCTGCGCCAAGAACTCGGTCAGCTCTACGCCGCCGTGCTGCACGGCGATGAAGACCCCCTCAGCGCCGTCAGCCCCCTCCCCATTCAGTACCGCGACTTCAGCATGTGGCAGCGCCGACAGCAAGTCGCCGAGCACGACCGACAGCTGCAGTACTGGCGAAAGCAGCTCGCTGACTGTTCGCCCGCCAAACTGCCCACCGATTTCCCCCGACCCCCCCTCCTGTCGGGCGATGCTGGCTCGGTCCCCGTCGAGATCAGCGGCGAGCTCTTTCAGAAGCTCCACCGCTTCTGCAACGTCACGAGCACCACCCCTTTTGCCGTCCTCCTGGCCGCTTTTCGCGCCGCCCACTACCGCCTCACGGGTGTGGATGACGCCGTCGTCGGCACCCCTATCGCCAACCGAAACCGCCCCGAGCTCGAGCGCCTCATTGGTTTCTTCGTCAACACGCAGTGCATGCGCATCACCGTCGACGACGATGATACCTTTGAGGGTCTCGTCCGCCAAGTCCGCCGCACCACGACCGAGGCCTTCGAGAACGAAGATGTGCCCTTTGAACGAGTCGTCTCGGCCATGCTCCCCGCTGGCGGTGGTTCGCGAGACCTGTCGCAGACCCCCCTCGCTCAGCTCATCTTCGCCGTCCACAGCCAAGAGAACCTCGGCAAGTTCGAGCTCGAGGGCCTGGAGAGCGAGCCCGTCGCCAACAAGGCCTACACCCGCTTCGACGCCGAGTTCCACCTCTTTCAGACCCGCGACGGCCTCAACGGCTACCTCAACTTCGCCGCCGAGCTCTTCAAGCTCGAGACCATGCAGAATGTCGTCAGCGTCTTTCTCCAAATCCTCCGACACGGCCTCGAGCAGCCCAAGAGCCTCATCAGCGTCCTCCCCCTGACCGACGGTCTCAAGGAACTGGACAGCATGGGCCTCCTCAAGATCCATCGCGGCCTCGAGTATCAGCGAGACTCGAGCCTGGTGGACATCTTCCGAAGCCAAGTCGCCACCTGCCCCGACACCATCGCCGTCATTGACAGCAGCGCCCGCCTCACCTACGCTCAGCTCGACCATCAGTCGAATCTCCTCGAGGCCTGGATTCGACGCAAAGGCCTCCCCGCCGAGAGCCTGGTCGGCGTCCTCAGCCCCCGCAGCTGCGAGACCATCATCGCCTTCCTCGGCATCCTCAAGGCCAACCTGGCCTACCTCCCCCTCGACCCCAAGAGCCCCGTCAGCCGAATGCGCGACGTCCTCAGCGACCTCCCCGGCCACACCATCATCCTCCTCGGCAGCGATGTCGCCGCCCCCGACCTCGAGCTCCCCTGCCTGGAACTCGTCCGCATCTCGGACGCCCTCAAAAGCGGCGCTAGCGCCGTCAATGGCTCGGAGACCACCGACCTCAGCGCCCCTAGCGCTAACTCGCTCGCCTATGTGCTCTACACGAGCGGTAGCACCGGCCGCCCCAAGGGCGTCATGGTCGAGCATCGCGCCATCGTGCGCCTCGTCCAACGCGGTGTCATCCCCAACTTCCCCCCCCTCCGCGGTGCCATCATGGCCCACCTCTTCAACACCGTCTTCGACGGCGCCACCTACGAGATCTTCCTCATGCTCCTCAACGGCGGCACCCTCGTCTGCATCGACTACCTCACGACCCTCTCGCCCAAGGCCCTCGAAACCGTCTTCCTCCGCGAGGGCATCAACTGCGCCATCATGACCCCCGCCCTGCTCAAGCTCTACCTCGCCAACGCCCGAGACGGCCTCAAGGGCCTGGACATGCTCATGGTCGCTGGCGATCGCTTCGACCCCCAAGACGCTGTGGAGGCTCAGACCCTCGTCCGAGGCGACTGTTACAATGCCTACGGCCCCACCGAGAACGGCGTCATGAGCACCCTCTACAAGATCGACACGAGCGACAGCTTCATCAATGGTGTGCCCCTCGGCCGCGCCATCGACAACAGCGGCGCTTACATCACCGACCCTAATCAGCAACTCGTCGGTCCCGGTGTGCTCGGCGAGCTCATCGTGACCGGCGATGGCCTCGCCCGCGGTTACACCGACCCCGCCCTCGATCGCGACCGCTTCGTCCAAGTCGTCATCAACGGCGAGAGCGTGCGCGCTTACCGAACCGGCGACCGAATGCGCTACCGCGCTGGCCAAGACTGCCTCTTCGAGTTCTTCGGCCGCATGGACTTTCAGTTCAAGATCCGCAGCAATCGAATCGAGAGCGCCGAGGTGGAGGCTGCCATTCTCAGCCACCCCCTGGTCCGCGATGCCGCCATCGTGGTCGTGGGCGTCCAAGAGGAGCAAGAGCCCGAGATGGTCGGCTTTGTCGTCGCCGCCGACGACGCCGTCGAGCAAGAGGCCACCGACAACCAAGTCGAGGGCTGGCAAGAGCTCTTCGAAAGCAGCATGTACAACGGCATCGACGCCATCAGCCCTAGCGCTCTCGGCAAAGATTTCACCGGCTGGACGAGCATGTACGACGGCAGCGAGATTGACAAGAGCGAGATGCAAGAGTGGCTCGACGACACCATCCACACGCTCCGAGACGGCCATGTCCCCGGCCACGTCCTCGAGATCGGCACCGGCACCGGCATGATCCTCTTCAACCTCGGCAGCGTCGAGAGCTACGTCGGCCTCGAGCCCACCAAGAGCGCCGTCGAGTTCGTCAACAAGGCCATCAAGACCCTCCCCAACCTCGCTGGCCGCGCCGAAGTCCACACCGGCACGGCCACCGACATCGATCAGCTCAGCGGCCTCCGCCCCGACCTCGTCATCCTCAACAGCGTCGTGCAGTACTTCCCCACCGTCGAGTACCTCACCCGCGTCGTCGACGCCCTCGTCCGAATCCGAGGCGTCAAGCGCCTCTTCTTCGGCGACGTCCGCAGCCAAGCCCTCCACCGACAGTTCCTCGCTGCTTGCGCCATGCACGCTCTCGGTAAGACCGCTACCCGCGATGACGTCCGACGCTACATGGCCGAACGCGAGGAGCGCGAGGAAGAGCTCCTCGTCGAGCCCGCCTTCTTCACCGCCCTCATGAACCGCCACCCCAACCTCATTCAGCACGTCGAGATCCTCCCCAAGAACATCCGCGCCACCAACGAGCTCAGCGCCTACCGCTATGCCGCTGTGGTCCACCTGCGCGACCCCGAGTCGGCTGCCCGCCCTGTCTACCCCATTGCTGCCGATGACTGGGTGGACTTCCAAGCCTCGCAGATGCGCTCGGACGTGCTCCGAGAGTATCTCCGCCTCAGCGCCGGTGCCGACACCGTCGCCGTCTGCAACATCCCCTACGAGAAGACCATCTTCGAGCGACTCATCGTCGAATCGCTCGACGACAATACGGGCTCGGACGCTCCTCAGAGCCGCCTCCATGGTCGCAGCCTCGATGGTGCCCCTTGGATTTCGGCTGTGCGCAGCGACGCCGAGAGCCGAGCCTCGCTCAGCGTCCCCGATCTCGTCCAACTCGCCGCCGAGTCGGGTTTTCAAGTGCAAGTCAGCGCCGCCCGACAGTGGAGCCAAAGCGGCGCCCTGGACGCTGTCTTTCACCGCCGCCACGCTAGCTCGTCGCAGCCCACCATGCGCACCCTCTTTCAGTTTCCCGACGATAACGCCCTGCGCGCTAGCGCCACCCTGACCAACCGACCTCTGCAGCGCCTCCAACGCCGACGCGTGGCTGCTCAAATCCGCGAGCGCCTGCAGACCCTGGTCCCTAGCTACATGATCCCCGCCAAGATCGTCGTCCTCGATCAAATGCCCCTCAACGCCAACGGTAAGGTCGACCGCAAAGAACTCGCCCGCCGAGCCCGCACGACGACCATGACCAAGAAAAAGAAGCCTCAGCGCCTCGCCTCGGAGCCCGCCTGCCCCATCAGCGACATCGAGGTCGCCCTCTGCGAAGAGGCCACGGCCACCTTCGGCATGCAAGTCGGCATCAGCGACCATTTCTTTAAGCTGGGCGGTCACAGCCTCCTCGCCACCAAACTCATCAGCCGCGTCGGCGACCGCCTCAAGGCCCGACTCACCGTCAAAGATGTCTTCGACCACCCCATCTTCAGCGAGCTCGCCATCGTCATCCGCGAGGGCCTCCAAAACGTGGTCCCCGTGGCCCTGAACGGTGGCGGTCAAGCCAAACAAGGTAGCGCCGGCGTCGTCGCCCCCCGCAACGAGATGGAGACCATGCTCTGCGAGGAGTTCGCCAACGTCCTCGGCATGGACGTCGGCGTCACCGACAACTTCTTTGATCTCGGCGGCCACAGCCTGATGGCCACCAAACTGGCCGCCCGAATCGGCCGCCGCCTCAACACCACCATCAGCGTCAAAGAGGTCTTCGAGCACCCCATCGTCTTTCAGCTCGCCAACAGCCTCGAGCTCGGTCAGCTCGAAAGCGACCGCGTCAAGCACACCATGCTCGCCGACTACACCGCCTTTCAGCTCCTCAGCGTCGAGGACCTCCAAGGCTTCCTGCAGAATGAAATCAGCCCTCAGCTCGAGTGCGCCCATGGCGGCATCCAAGACGTCTACCCCGCCACCCACATGCAGAAGGCCTTCCTCTGCGACGCTAGCACCGGCCACCCCAAGCCCCTCGTCCCCTTCTACATCGACTTCCCCCCCGATAGCGACTGCAGCACCCTCGTCGAGGCCTGCAGCAGCCTGGTGAAGCGCTTCGACATGTTCCGCACCGTCGTGGTCGAGGCTGCCGGCGAGCTCTACCAAGTCGTCCTCGAGCACTTCGACCTGCAGATCGACGTCGTCGAGACCGAGGAGAACGTCCACGCCGCCACGAACGACTTCGTCGACCGCATTCTCGAGGTCCCCGTCCACCTCGGTCAGCCCCTCATTCAGTTCACCATCCTCAAGCAAGCTAGCAGCGTCCGCGTCCTGCTCTGCCTCAGCCACGCCCTCTACGACGGCCTCAGCCTCGAGCACGTCGTCCGCGATCTCCACATGCTCTACAAGGGCCGCAGCCTCCTCCCCGCTAATCAGTTCAGCCGCTACATGCAGTACATGGACCACACCCGCAAGGCCGGCTGCGACTTCTGGCGCGACGTCATCCAAGACACGCCCATCACCGTCCTGGGTCACGTCGACGCTGGTGGTCGAGAGCTCGAGGTGGAAGCCGCCCGAACCCTCCACGCCACCAAGATCATCAGCATCCCCCTGCAAGCCGTCCGCAGCAGCATTATCACCCAAGCCACCGTCTTCAACGCCGCCTGCGCCCTGGTGCTCAGCCGAGAGACCGGTGCCAAGGACGTCGTGTTCGGTCGCATCGTCAGCGGCCGCCAAGGCCTCCCCGTGAGCTGGCAGAACATCGTCGGTCCCTGCACCAACGCCGTCCCCGTCCGCGCCCGCATCATCGATGATGATGACGACAACCACCGACAGATGCTCCGCGACATGCAAGATCAGTATCTCCTCAGCCTCCCCTTCGAAACCCTCGATTTCGACGAGGTCCGCCGCTCGTGCACCAACTGGCCCGCCACCGCCAACAACTACGCCTGTTGCGTCACCTACCACGACTTCAGCTACCATCCTGAGAGCGAAATGGAGCAGCAGCGCGTGGAGATGGGCGTCCTGGCCCGCAAGGACGCTCTGCTGAAGGAGGAGCCTGTGTACGACCTCGGCATCGCCGGCGAGGTGGAGCCCGATGGCGTCCACCTCCAAGTCACCGTCGTCGCCAAGACCCGCCTCTTCTCGGAAGAGCGCGCCGCTTACCTCATGGAAGAGGTCTGCCGCCTCTTCGAGAGCCTCAATAGCGCCCTCTGA

# Other Genetic transformation methods of *Emericellopsis* sp. XJ1056

Specific methods of endonuclease-mediated protoplast transformation (REMI) (Wang et al. 2009), electroshock transformation followed by previous reports (Gutiérrez et al. 2011) and *Agrobacterium tumefaciens*-mediated transformation (ATMT) were followed by previous reports (Chen et al. 2013).

# Supplementary Figures and Tables


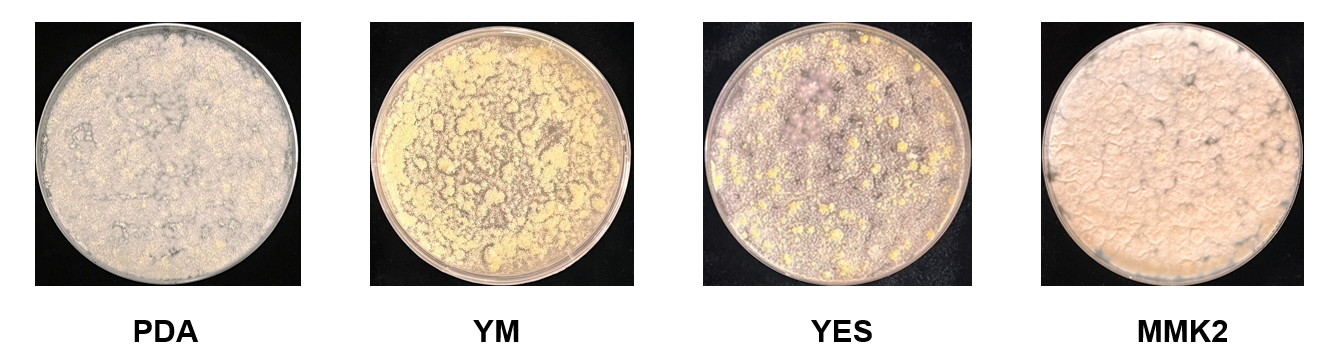


## **Figure S1-1** The morphology of the *Emericellopsis* sp. XJ1056 wild type cultured on different media for 10 days.


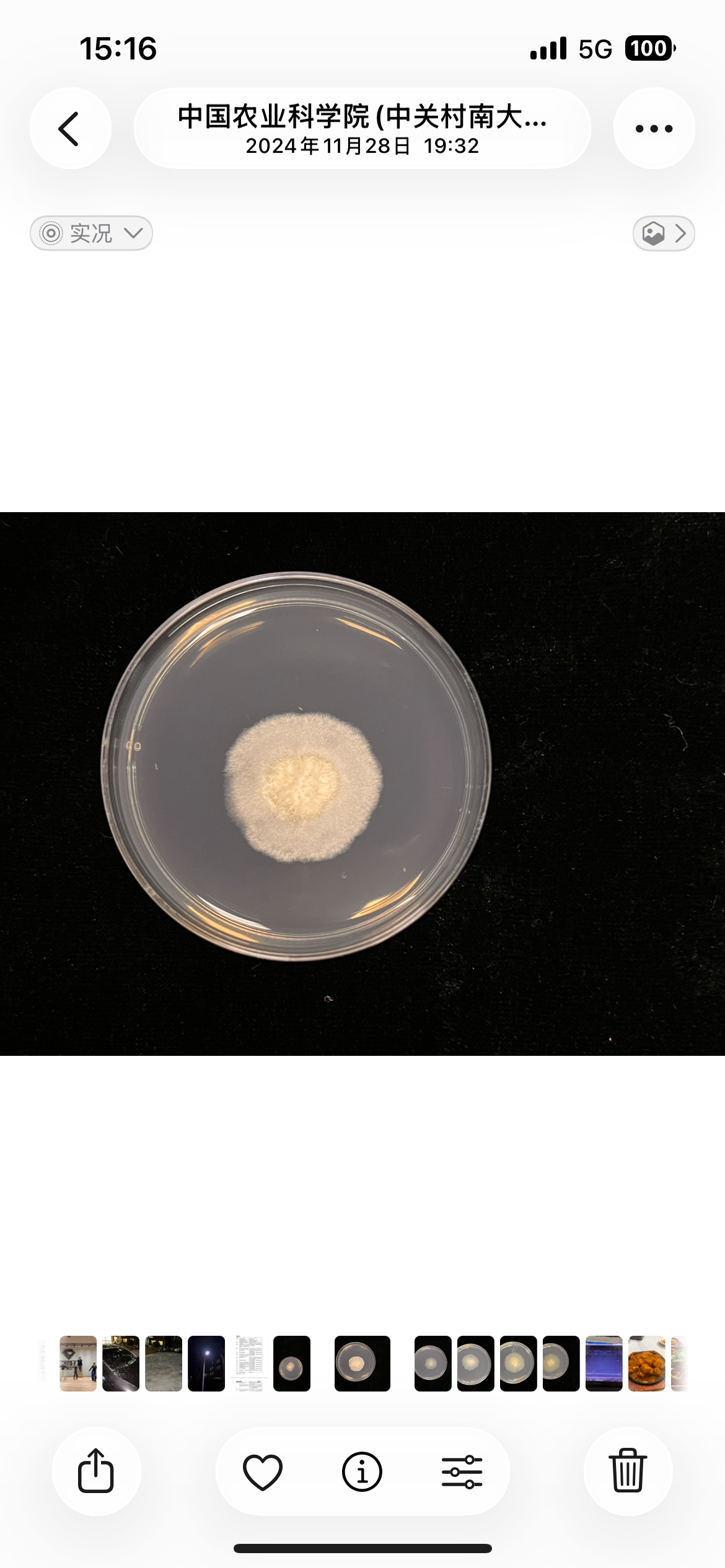


## **Figure S1-2** No-antibiotic control of the *Emericellopsis* sp. XJ1056 wild type.


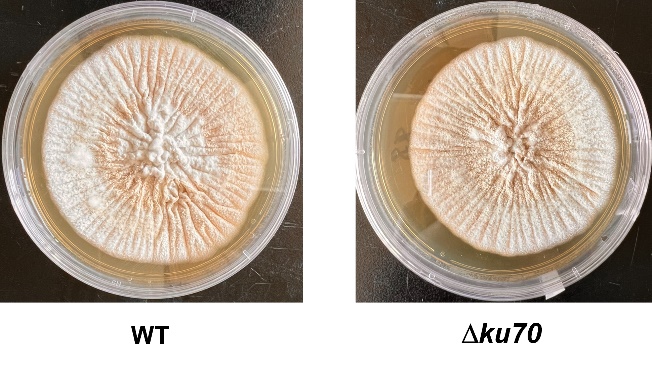


## **Figure S1-3 T****he morphology of the** ***Emericellopsis* sp. XJ1056 wild type (WT) and the** Δ*ku70* strain **cultured on YES medium for 10 days.**

**
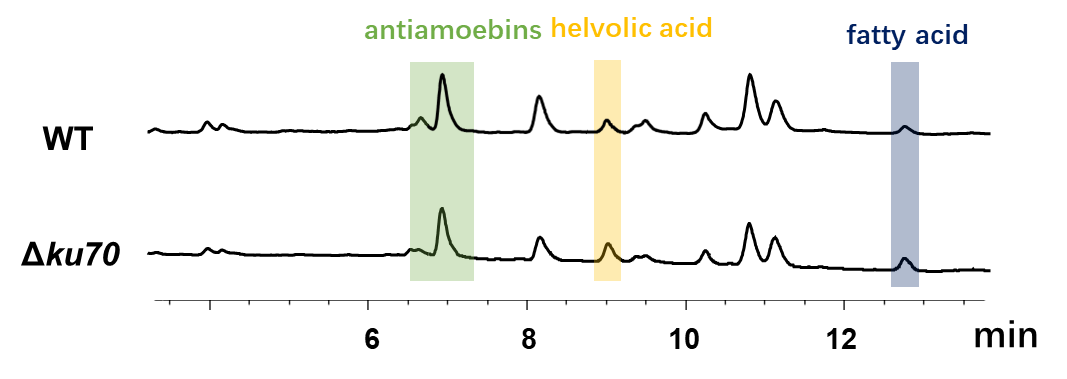
** Figure S2-1 Chromatograms of the crude extracts from the *Emericellopsis* sp. XJ1056 wild-type strain (WT) and the Δ*ku70* strain


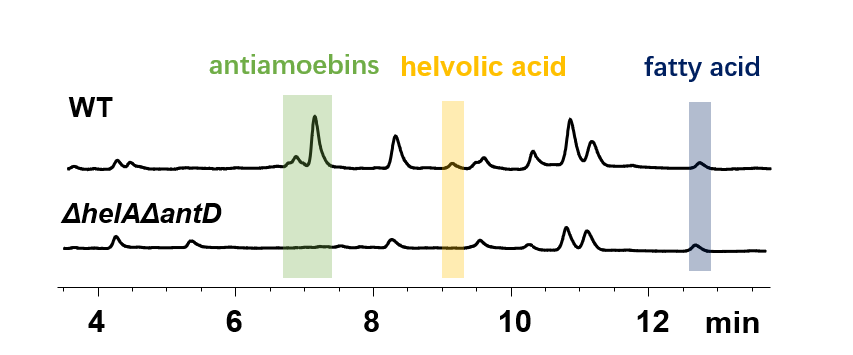


## Figure S2-2 The secondary metabolites produced by the *Emericellopsis* sp. XJ1056 wild type (WT) and Δ*helA*Δ*antD* strain after being cultured on YES medium for 10 days. The secondary metabolites were almost non-existent after knocking out *helA* and *antD*.


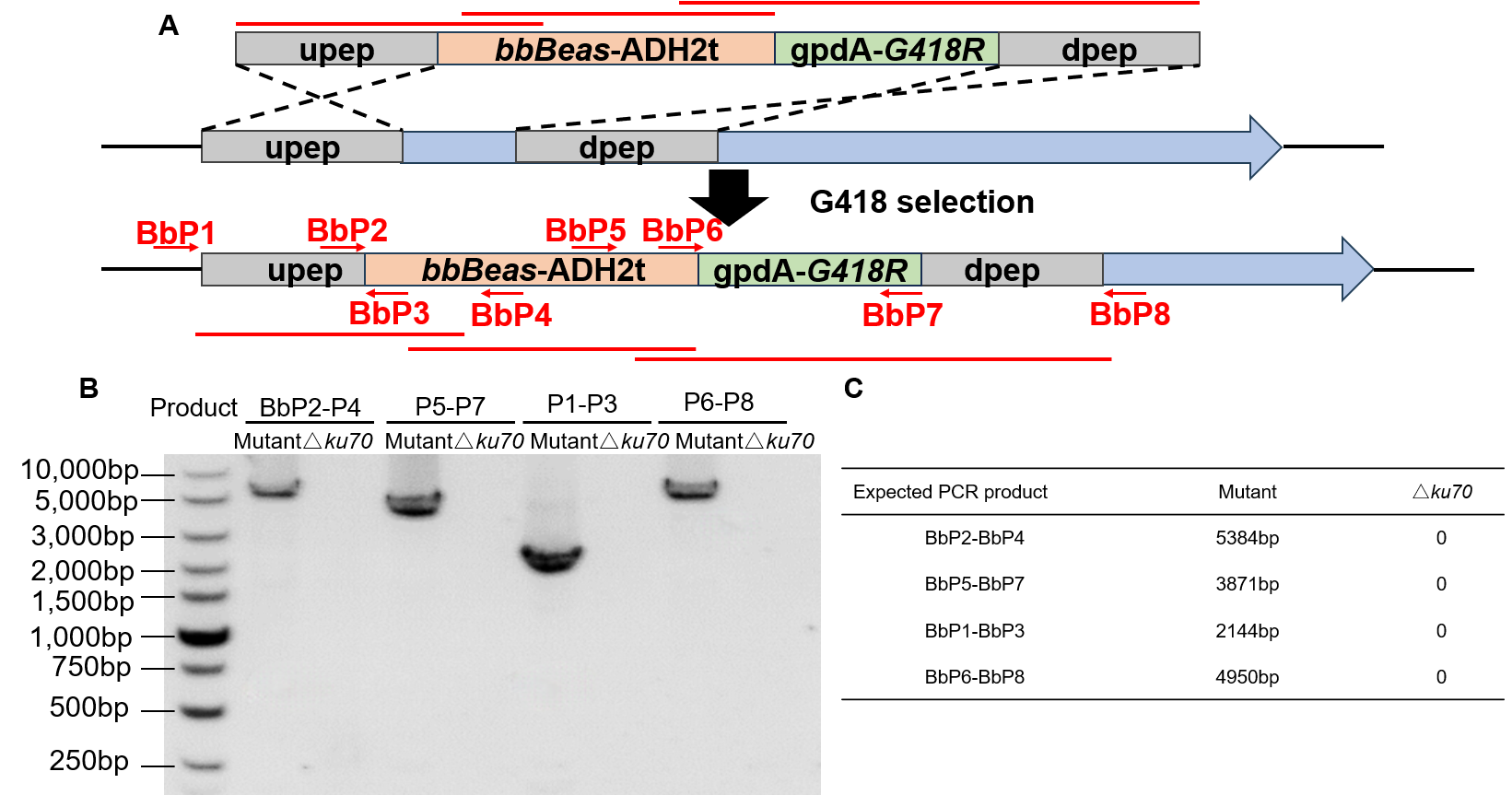


## **Figure S3** Construction and transformation of *bbBeas* insertion strain

(A) Schematic of *bbBeas* insertion. (B) PCR products for representative isolates of the Δ*ku70* strain (Control) and the Δ*ku70-bbBeas* mutant. (C) The expected lengths of the PCR products amplified by the appropriate primers using the Δ*ku70* strain or the mutant Δ*ku70-bbBeas* genomic DNA as the template.


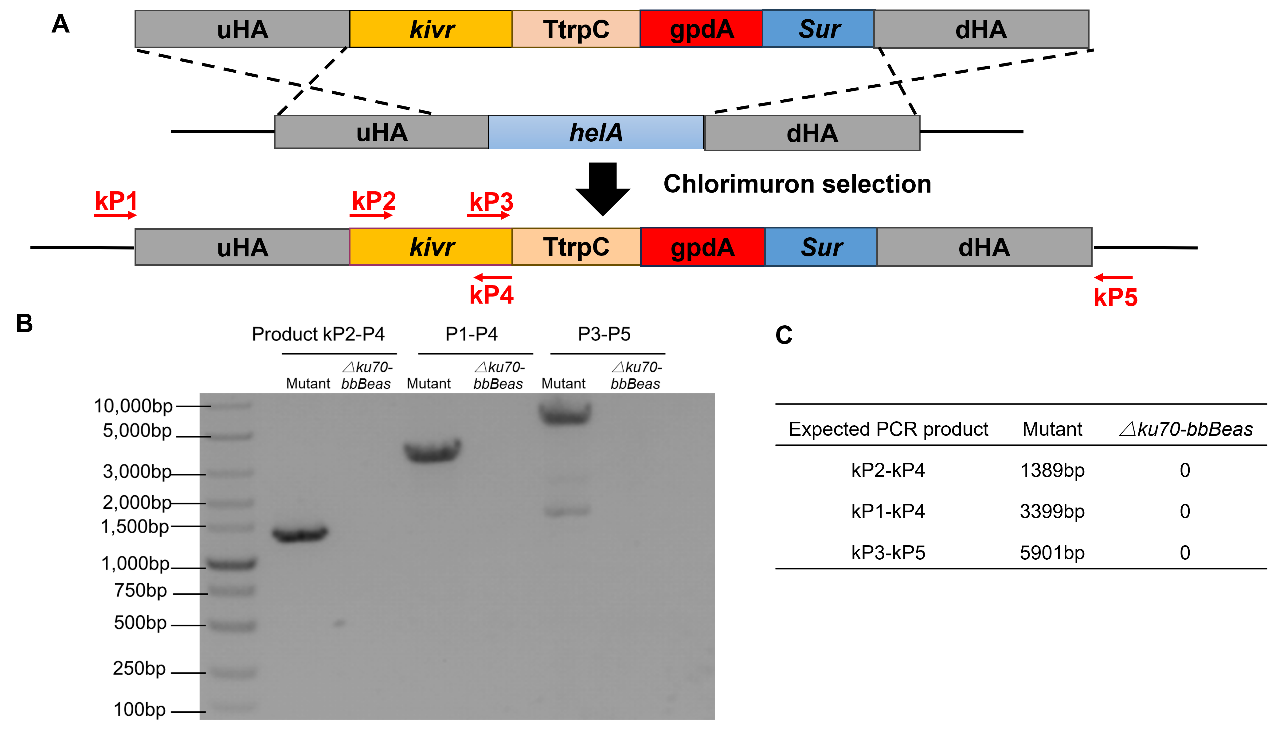


## **Figure S4** Construction and transformation of *kivr* insertion strain

1. Schematic of *kivr* insertion. (B) PCR products for representative isolates of the Δ*ku70-bbBeas* strain (Control) and the Δ*ku70-bbBeas-kivr* mutant. (C) The expected lengths of the PCR products amplified by the appropriate primers using the Δ*ku70-bbBeas* strain or the mutant Δ*ku70-bbBeas-kivr* genomic DNA as the template.


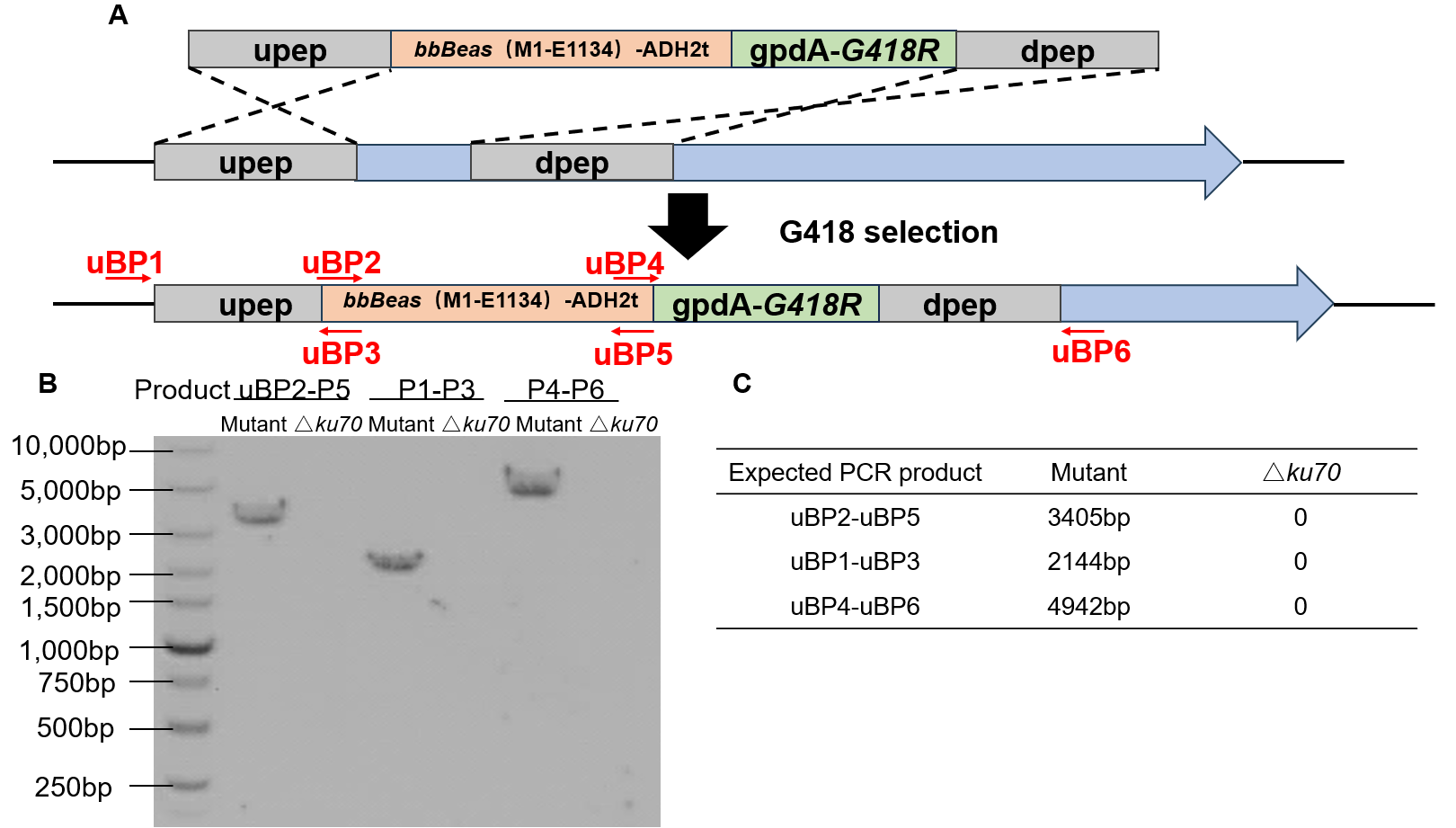


## **Figure S5** Construction and transformation of the upstream unit of *bbBeas* insertion strain

(A)Schematic of *upbbBeas* (M1-E1134) insertion. (B)PCR products for representative isolates of the Δ*ku70* strain (Control) and the Δ*ku70-upbbBeas* mutant. (C)The expected lengths of the PCR products amplified by the appropriate primers using the Δ*ku70* strain or the mutant Δ*ku70-upbbBeas* genomic DNA as the template.


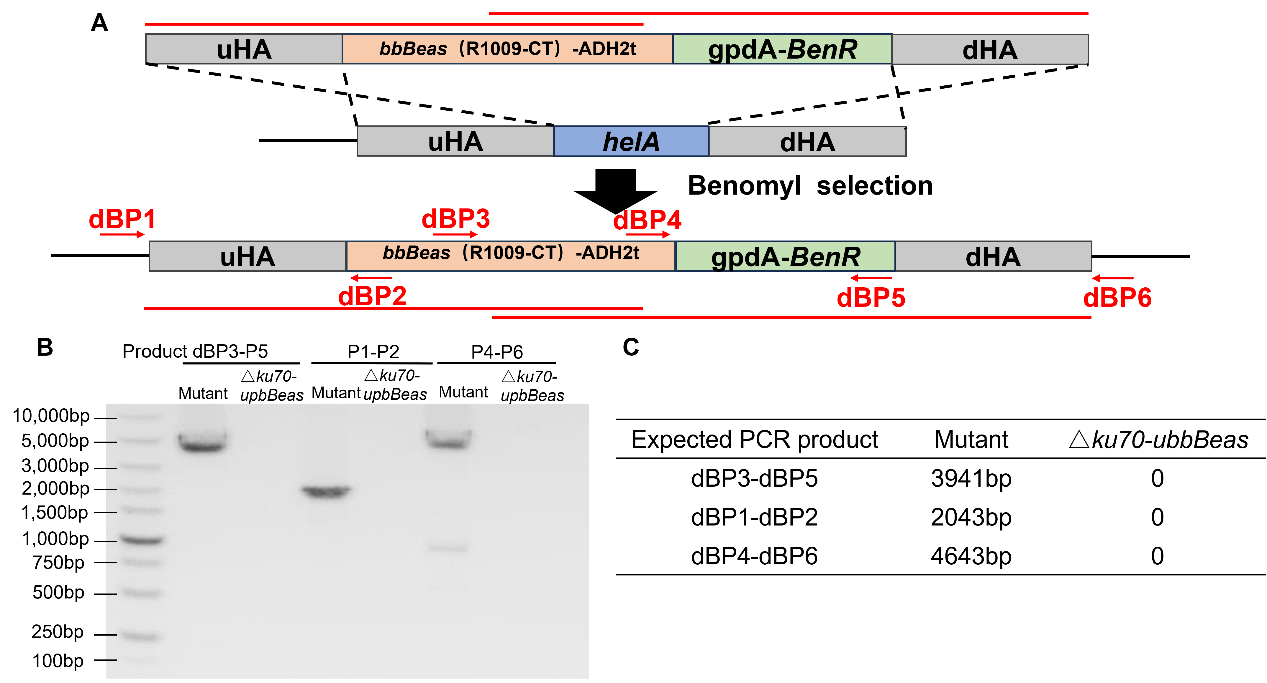


## **Figure S6** Construction and transformation of the downstream unit of *bbBeas* insertion strain

(A)Schematic of *downbbBeas* (R1009-CT) insertion. (B)PCR products for representative isolates of the Δ*ku70-upbbBeas* strain (Control) and the Δ*ku70-udbbBeas* mutant. (C)The expected lengths of the PCR products amplified by the appropriate primers using the Δ*ku70-upbbBeas* strain or the mutant Δ*ku70-udbbBeas* genomic DNA as the template.


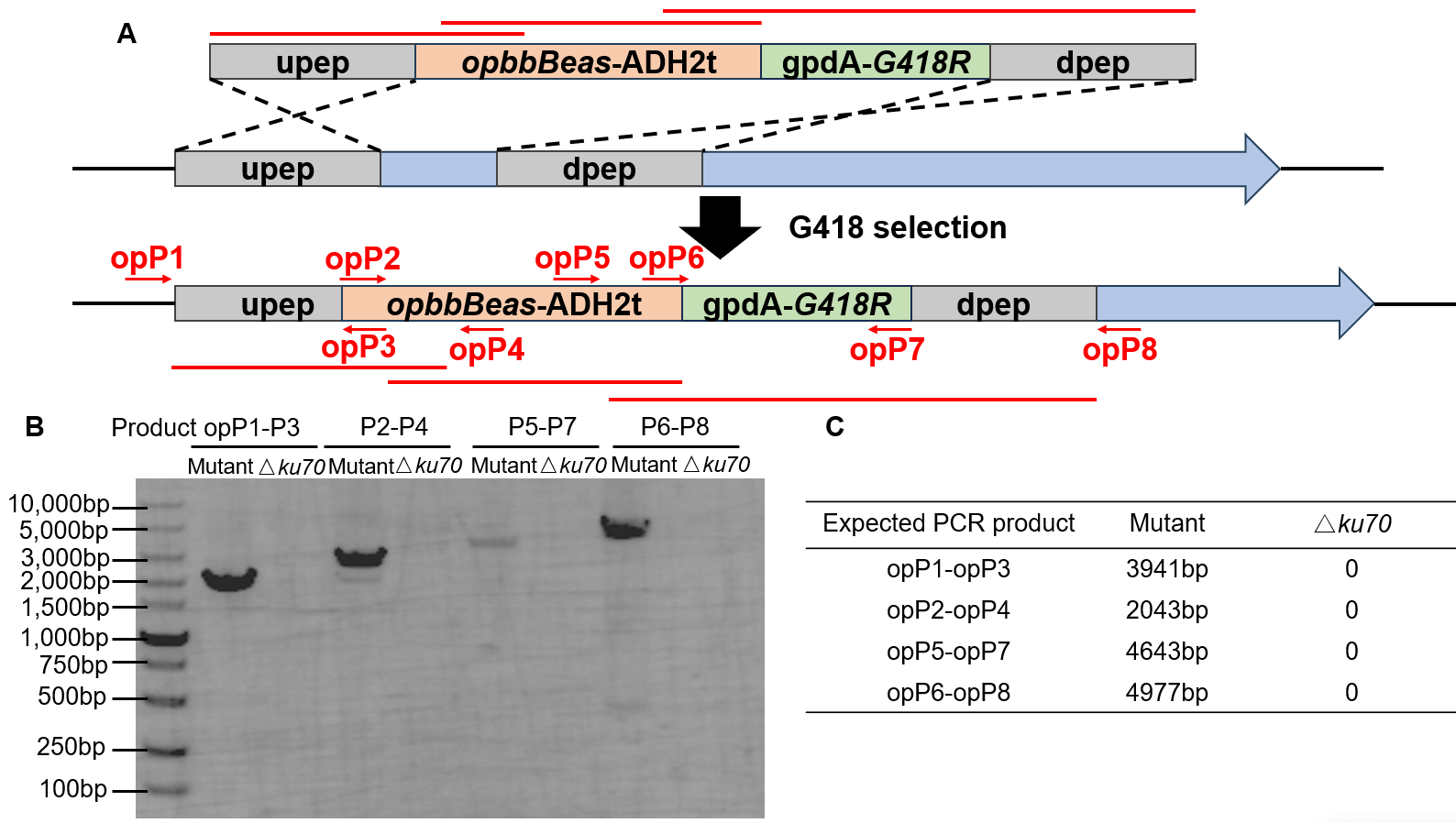


## **Figure S7** Construction and transformation of optimized *bbBeas* insertion strain

1. Schematic of *opbbBeas* insertion. (B) PCR products for representative isolates of the Δ*ku70* strain (Control) and the Δ*ku70-opbbBeas* mutant. (C) The expected lengths of the PCR products amplified by the appropriate primers using the Δ*ku70* strain or the mutant Δ*ku70-opbbBeas* genomic DNA as the template.

## Figure S8 The yield of beauvericin of the strain Δ*ku70-bbBeas-kivr* on different ratios of rice to yeast extract

^***^^*^: P value<0.0001 compared with the group 3:1


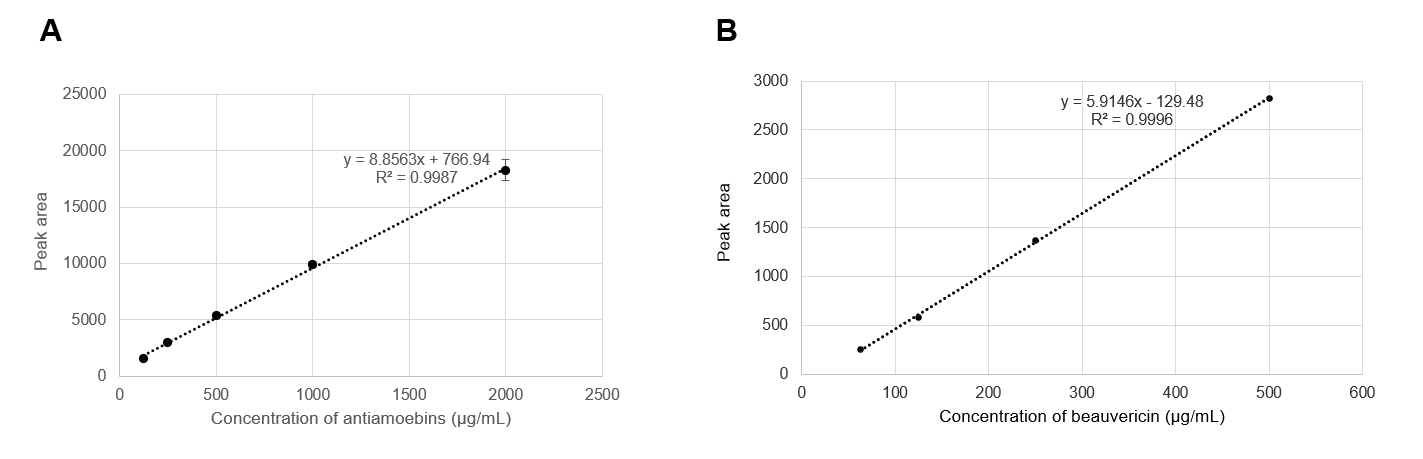


## **Figure S****9** The standard curve of antiamoebins and beauvericin.

(A)The standard curve of antiamoebins. (B)The standard curve of beauvericin.


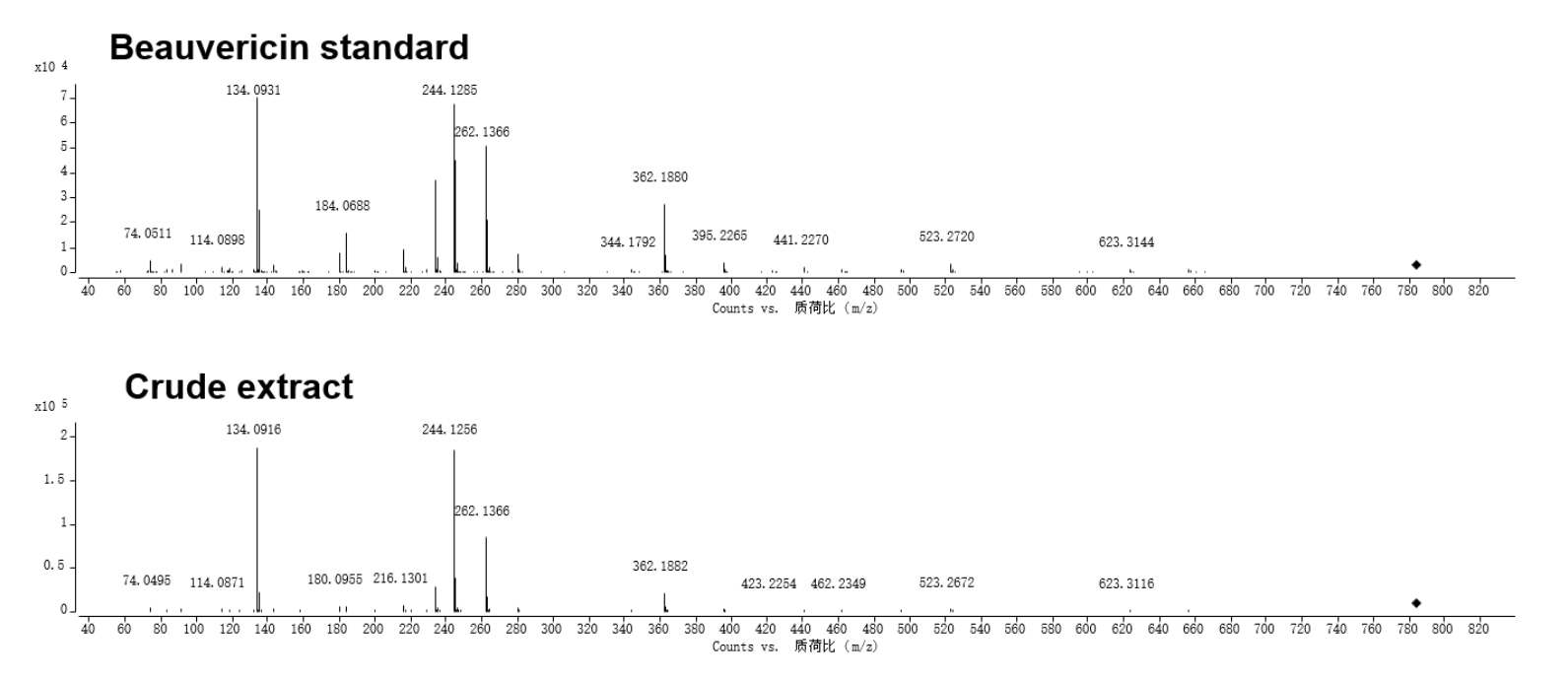


## **Figure S10** HRESIMS spectrum of beauvericin and crude extract of the strain Δ*ku70-opbbBeas-kivr*

## **Table S1** Strains and plasmids used in this study.

| Names | Description | References or sources |
| --- | --- | --- |
| Strains | | |
| *Emericellopsis* sp. XJ1056 | Strains producing large number of antiamoebins, wild type | This study |
| Δ*ku70* | Strains lacking of *ku70* gene | This study |
| Δ*ku70-bbBeas* | Δ*ku70* derivative, inserted the whole gene *bbBeas* using the promoter of *antD* | This study |
| Δ*ku70-bbBeas-kivr* | Δ*ku70-bbBeas* derivative*,* inserted the *kivr* using the promoter of *helA* | This study |
| Δ*ku70-opbbBeas* | Δ*ku70 derivative, inserted the gene opbbBeas using the promoter of antD* | This study |
| Δ*ku70-opbbBeas-kivr* | Δ*ku70-opbbBeas* derivative*,* inserted the *kivr* using the promoter of *helA* | This study |
| Plasmids | | |
| pAg1-H3 | PtrpC-HygR (Hygromycin-resisitent gene)-TtrpC, T-DNA repeat, oriV, KanR, trfA | (Yin et al. 2021) |
| pAg1-ku70 | pAg1-H3 derivative, inserted the hygromycin-resistant gene h*ygB* and its promoter PtrpC and terminator TtrpC, upstream arm uku70 and downstream arm dsku70{pAg1-uku70-PtrpC-hygB-dku70} | This study |
| pAg1-bbBeas | pAg1-H3 derivative, inserted the upstream arm upep and downstream arm dpep, *bbBeas* and its terminator ADH2t, geneticin -resistant gene *G418R* and its promoter gpdA {pAg1-upep-bbBeas-ADH2t-gpdA-G418R-dpep} | This study |
| pJET | Vector backbone, containing ori, *AmpR* | (Wang et al. 2022) |
| pJET-kivr | pJET derivative, inserted the upstream arm uHA and downstream arm dHA, *kivr* and its terminator TtrpC, the chlorsulfuron-resistant gene *Sur* and its promoter gpdA {pJET-uHA-kivr-TtrpC-gpdA-Sur-dHA} | This study |
| pYTR | Vector backbone, containing AMA1 ori for *Aspergillus*, gpdA promoter, riboB, *AmpR*, URA3 | (Xie et al. 2021) |
| pUC-Cas-neo | Vector backbone, containing gpdA promoter, nuclease gene *cas9*, terminator TtrpC, geneticin -resistant gene *G418R* | (Luo et al. 2023) |
| pDHt-ben-gpdA | Vector backbone, containing gpdA promoter, benomyl-resistant gene Ben*R* | (Chen et al. 2015) |
| pK2-Sur | Vector backbone | (Chen et al. 2022) |
| pJET-upbbBeas | pJET derivative, inserted the upstream arm upep and downstream arm dpep, the upstream unit of *bbBeas* (M1-E1134) and its terminator ADH2t, geneticin -resistant gene *G418R* and its promoter gpdA {pJET-upep-bbBeas(M1-E1134)-ADH2t-gpdA-G418R-dpep} | This study |
| pJET-downbbBeas-1 | pJET derivative, inserted the upstream arm uHA, the first part of the downstream unit of *bbBeas*(R1009-CT) {pJET-uHA-bbBeas(R1009-CT)-1} | This study |
| pJET-downbbBeas-2 | pJET derivative, inserted the downstream arm dHA, the second part of the downstream unit of *bbBeas* (R1009-CT) and its terminator ADH2t, benomyl-resistant gene Ben*R* and its promoter gpdA {pJET-bbBeas(R1009-CT)-2-gpdA-BenR-dHA} | This study |
| pJET-opbbBeas-1 | pJET derivative, inserted the upstream arm upep, the first part of the optimized *bbBeas* {pJET-upep-opbbBeas-1} | This study |
| pJET-opbbBeas-2 | pJET derivative, inserted the second part of the optimized *bbBeas*{pJET-opbbBeas-2} | This study |
| pJET-opbbBeas-3 | pJET derivative, inserted the downstream arm dpep, the third part of the optimized *bbBeas* and its terminator ADH2t, geneticin -resistant gene *G418R* and its promoter gpdA {pJET-opbbBeas(R1009-CT)-3-gpdA-BenR-dHA} | This study |

## **Table S2** Primers used in this study.

| Peimers | Primer sequence | application |
| --- | --- | --- |
| pAg1-F1 | GCAAGCTTCGTGACTCCCT | To amplify the linear vector pAg1 |
| pAg1-R1 | GGCCCATCGATGATCAGG |  |
| pAg1-Sku70-F | gcctgatcatcgatgggccatacatgtgctccgtcaagggattgg | To amplify the upstream arm uku70, the underlined sequences are homologous arms with pAg1-H3 |
| sku70-up-R | aggtacgcgcaggtgtttgc |  |
| sku70-up-PtrpC-F | cacctgcgcgtaccttcgacagaagatgatattgaaggagcac | To amplify the hygromycin -resistant gene and its promoter PtrpC and terminator TtrpC, the underlined sequences are homologous arms with upstream arm uku70 |
| TtrpC-R | aagaaggattacctctaaacaagtgtac |  |
| TtrpC-sku70-down-F | gaggtaatccttcttgccctatggcagaagctcctg | To amplify the downstream arm dku70, the underlined sequences are homologous arms with terminator TtrpC and pAg1-H3 respectively. |
| pAg1-sku70-down-R | agtcacgaagcttgctccaacgaagattatcgacctcagccg |  |
| pAg1-upep-F | gcctgatcatcgatgggcctcggatacatttcatcgttggg | To amplify the upstream arm upep, the underlined sequences are homologous arms with pAg1-H3 and *bbBeas.* |
| Bbae-upep-R | ttttgagcggctccattttgaaatctgtatagtcaagtcgtagtc |  |
| Bbae-1-F | atggagccgctcaaaaatgtcaatactg | To amplify the *bbBeas* and its terminator ADH2t, the underlined sequences are homologous arms with the promoter gpdA. |
| gpdA-ADH2t-R | aaatcaattcaccggagtagggcgaattgggtaccctc |  |
| gpdA-F | actccggtgaattgatttgggtgacg | To amplify the promoter gpdA, the underlined sequences are homologous arms with the geneticin -resistant gene *G418R.* |
| G418-gpdA-R | catcttgttcaatcattgtttagatgtgtctatgtggcggggtaatg |  |
| G418-F | atgattgaacaagatggattgcacgc | To amplify the geneticin -resistant gene and its terminator |
| G418-R | tctagagaataggaacttcggaataggaac |  |
| dpep-F | tgcatcttccgcgcttctccc | To amplify the downstream arm dpep |
| dpep-R | tacggatcctgttctcttgggcg |  |
| pJET-F1 | CGACTCACTATAGGGAGAGCGGC | To amplify the linear vector pJET |
| pJET-R1 | AAGAACATCGATTTTCCATGGCAG |  |
| pjet-uHA-F | gtttttcagcaagacatgtcaaaggggacatgctctcc | To amplify the upstream arm uHA, the underlined sequences are homologous arms with the pJET and *kivr* respectively |
| kivr-uHA-R | atgctccggagatggcattgtgacagttactgagaatgtggacagaatg |  |
| kivr-F | atgccatctccggagcatcc | To amplify the *kivr,* the underlined sequences are homologous arms with the terminator TtrpC |
| TtrpC-kivr-R | ttcagtaacgttaagtggttacaacagccttctcgccagttcc |  |
| gpdA-F | ACTCCGGTGAATTGATTTGGGTGACG | To amplify the promoter gpdA, the underlined sequences are homologous arms with the chlorsulfuron-resistant gene *Sur* |
| Sur-gpdA-R | gccaacagtacgaagcattgtttagatgtgtctatgtggcggggtaatg |  |
| SurcDNA-F | atgcttcgtactgttggccg | To amplify the chlorsulfuron-resistant gene *Sur* |
| SurcDNA-R | ttaaccgtgcaggccattcgtc |  |
| dHA-F | tgtggaatatttacacgcaatccatttacgttg | To amplify the downstream arm dHA, the underlined sequences are homologous arms with the pJET |
| pjet-dHA-R | atcttctagaaagataggcttttgcgacagtcttcaaagc |  |
| pjet-upep-F2 | gtttttcagcaagatcggatacatttcatcgttggg | To amplify the upstream arm upep, the underlined sequences are homologous arms with pJET vector and *bbBeas* respectively |
| Bbae-upep-R | ttttgagcggctccattttgaaatctgtatagtcaagtcgtagtc |  |
| Bbae-1-F | atggagccgctcaaaaatgtcaatactg | To amplify the upstream unit of *bbBeas* |
| BbaeE1134-R | TCActcaacgggtccgtcccaag |  |
| BbaeE1134-ADH2T-F | gacggacccgttgagtgaaaacatgccttcacgatttatagtttccattatc | To amplify the ADH2t-gpdA-G418R-dpep, the underlined sequences are homologous arms with the upstream unit of *bbBeas* and pJET vector respectively |
| pjet-dpep-R6 | atcttctagaaagatCTCGAGACTGGGCCGAAATGTAG |  |
| pjet-uHA-F1 | gtttttcagcaagatcatgtcaaaggggacatgctctcc | To amplify the upstream arm uHA, the underlined sequences are homologous arms with pJET vector and the downstream unit of *bbBeas* respectively |
| BbaeR1009-uHA-R | gctgccgatagagcgcattgtgacagttactgagaatgtggacagaatg |  |
| BbaeR1009-F | ATGcgctctatcggcagcaagctc | To amplify the downstream unit of *bbBeas* and the ADH2t, the underlined sequences are homologous arms with the promoter gpdA |
| gpdA-ADH2T-R | aaatcaattcaccggagtagggcgaattgggtaccctc |  |
| gpdA-F | actccggtgaattgatttgggtgacg | To amplify the gpdA-*BenR*, the underlined sequences are homologous arms with the downstream dHA |
| dHA-Ben-R | gcgtgtaaatattccacattactcctcgccctcaagggg |  |
| dHA-F | tgtggaatatttacacgcaatccatttacgttg | To amplify the downstream dHA, the underlined sequences are homologous arms with the pJET vector |
| pjet-dHA-R | atcttctagaaagataggcttttgcgacagtcttcaaagc |  |
| pjet-upep-F | gtttttcagcaagatGTTGACGTTGCATAGGCTCTCGAG | To amplify the upstream upep, the underlined sequences are homologous arms with the optimized *bbBeas* |
| opBbae1-upep-R | gttcttgaggggctccattttgaaatctgtatagtcaagtcgtagtcgttg |  |
| opBbae1-F | ATGGAGCCCCTCAAGAACGTC | To amplify the first part of optimized *bbBeas* |
| opBbae1-R1 | ATGTTGTTGGCCTCGCTGAC |  |
| opBbae1-opBbae2-F | cagcgaggccaacaacattggtcgagccgtcggcgctCACTCGTGGATCGTCCATCCC | To amplify the second part of optimized *bbBeas,* the underlined sequences are homologous arms with the first part of optimized *bbBeas* |
| pjet-opBbae2-R2 | atcttctagaaagatCTGCTGTCAATGACGGCGATGG |  |
| pjet-opBbae2-F | gtttttcagcaagatCACTCGTGGATCGTCCATCCC | To amplify the second part of optimized *bbBeas,* the underlined sequences are homologous arms with the pJET vector |
| opBbae2-R2 | CTGCTGTCAATGACGGCGATGG |  |
| ku70hph-tF2（ku70P1） | cccgttgttgaacaggagtactgagg | For *ku70* knockout verification |
| ku70hph-tF1（ku70P2） | cgagattgccccaggactacgaatc |  |
| ku70hph-tR1（ku70P3） | caccaggcaaggccgactttg |  |
| ku70hph-tR2（ku70P4） | gacaattctggtgatggggtggcc |  |
| uCYP5150L8-tF1(BbP1) | ccaaaatctgtgtgctccgagatgc | For *bbBeas* insertion verification |
| upep-tF5(BbP2) | gggtcttgtacctgggaagaagc |  |
| Bbae-tR2(BbP3) | tggctgcccagtattgacatttttgag |  |
| Bbae-tR1(BbP4) | tctcactgccattgactgcgcttg |  |
| Bbae-tF1(BbP5) | ctcagactgttctactctggtcgag |  |
| Bbae-tF2(BbP6) | ctgtttgagagtctaaactcggctttgtga |  |
| G418-R(BbP7) | tctagagaataggaacttcggaataggaac |  |
| dCYP5150L8-tR1(BbP8) | caatattcagaaagctgtgcgtgaggc |  |
| uHAOSC-tF1（kP1） | acattttcgtcttggactctggatcctc | For *kivr* insertion verification |
| kivr-F（kP2） | atgccatctccggagcatcc |  |
| kivr-tF1（kP3） | ggaactggcgagaaggctgttg |  |
| kivr-R（kP4） | ttacaacagccttctcgccagttcc |  |
| uHAOSC-tR1（kP5） | ttgacacggggcatgaaaccagac |  |
| uCYP5150L8-tF1（uBP1） | ccaaaatctgtgtgctccgagatgc | For upstream unit of *bbBeas* insertion verification |
| Bbae-1-F（uBP2） | atggagccgctcaaaaatgtcaatactg |  |
| Bbae-tR2（uBP3） | tggctgcccagtattgacatttttgag |  |
| BbaeE1134-F（uBP4） | ttgggacggacccgttgagTG |  |
| BbaeE1134-R（uBP5） | TCActcaacgggtccgtcccaag |  |
| dCYP5150L8-tR1（uBP6） | caatattcagaaagctgtgcgtgaggc |  |
| uHAOSC-tF1(dBP1) | acattttcgtcttggactctggatcctc | For downstream unit of *bbBeas* insertion verification |
| BbaeR1009-tR2(dBP2) | ttgctctaggagcttgctgccg |  |
| BbaeR1009-tF1(dBP3) | aatgttggccgattacactgcgtttc |  |
| Bbae-tF2(dBP4) | ctgtttgagagtctaaactcggctttgtg |  |
| Ben-R(dBP5) | ttactcctcgccctcaagggg |  |
| uHAOSC-tR1(dBP6) | ttgacacggggcatgaaaccagac |  |
| uCYP5150L8-tF1(opP1) | ccaaaatctgtgtgctccgagatgc | For optimized *bbBeas* insertion verification |
| opBbae1-F(opP2) | ATGGAGCCCCTCAAGAACGTC |  |
| opBbae1-tR1(opP3) | GGCTGACCGGTGTTGACGTTC |  |
| opBbae2-tR1(opP4) | CTTCAGCCACCACGGTGATGTC |  |
| opBbae4-tF1(opP5) | ACCCACATGCAGAAGGCCTTC |  |
| opBbae4-tF2(opP6) | GCTTACCTCATGGAAGAGGTCTGCC |  |
| G418-R1(opP7) | tctagagaataggaacttcggaataggaacttcaaag |  |
| dCYP5150L8-tR1(opP8) | caatattcagaaagctgtgcgtgaggc |  |

The effects of *ku70* deletion, knockout location, the length of the transforming fragment, and the number of fragments on the homologous recombination efficiency of *Emericellopsis* sp. XJ1056.

## **Table S3** The homologous recombination efffciency

1. The homologous recombination efffciency after or before the *ku70* knocking out

|  | The total number of transformants | The correct number of transformants | Accuracy rate | Notes |
| --- | --- | --- | --- | --- |
| *ku70* knocking out | 32 | 3 | 9.4% | Before *ku70* knocking out |
| *kivr* insertion | 10 | 5 | 50% | After *ku70* knocking out |
| *antD* knocking out | 7 | 5 | 71.43% |  |
| *CYP5150L8* insertion | 19 | 14 | 73.68% |  |

1. The homologous recombination efffciency of different length of DNA fragments

| The length of DNA fragments | The total number of transformants | The correct number of transformants | Accuracy rate | Notes |
| --- | --- | --- | --- | --- |
| 5754bp | 7 | 5 | 71.43% | *antD* knocking out |
| 9173bp | 19 | 14 | 73.68% |  |
| 10002bp | 9 | 7 | 77.78% |  |

1. The homologous recombination efffciency of different positions

| The starting position of knocking out on Scaffold2(^th^ bp) | The total number of transformants | The correct number of transformants | Accuracy rate | Notes |
| --- | --- | --- | --- | --- |
| 123445 | 7 | 5 | 71.43% | *antD* knocking out |
| 140890 | 24 | 6 | 25% |  |
| 144226 | 9 | 6 | 66.67% |  |
| 147487 | 19 | 8 | 42.1% |  |

(D) The homologous recombination efffciency of number of DNA fragments

|  | The number of DNA fragments | The total number of transformants | The correct number of transformants | Accuracy rate | Notes |
| --- | --- | --- | --- | --- | --- |
| *antD* knocking out | 1 | 7 | 5 | 71.43% | Knocking out or insertion position all after the *antD* promoter |
| upstream of *bbBeas* insertion | 1 | 9 | 7 | 77.78% |  |
| *bbBeas* insertion | 2 | 21 | 13 | 61.9% |  |
| *PFSYN* insertion | 2 | 10 | 6 | 60% |  |

# References

Chen, L.; Yue, Q.; Zhang, X.; Xiang, M.; Wang, C.; Li, S.; Che, Y.; Ortiz-López, F. J.; Bills, G. F.; Liu, X.; An, Z., Genomics-driven discovery of the pneumocandin biosynthetic gene cluster in the fungus *Glarea lozoyensis*. *BMC Genomics* 2013, *14*, 339. doi: 10.1186/1471-2164-14-339

Chen, Y.; Duan, Z.; Chen, P.; Shang, Y.; Wang, C., The Bax inhibitor MrBI-1 regulates heat tolerance, apoptotic-like cell death, and virulence in *Metarhizium robertsii*. *Sci Rep* 2015, *5*, 10625. doi: 10.1038/srep10625

Chen, X.; Zhang, W.; Wang, J.; Zhu, S.; Shen, X.; Chen, H.; Fan, Y., Transcription factors BbPacC and Bbmsn2 jointly regulate oosporein production in *Beauveria bassiana*. *Microbiol Spectr* 2022, *10* (6), e0311822. doi: 10.1128/spectrum.03118-22

Gutiérrez, A.; López-García, S.; Garre, V., High reliability transformation of the basal fungus *Mucor circinelloides* by electroporation. *J Microbiol Methods* 2011, *84* (3), 442-6. doi: 10.1016/j.mimet.2011.01.002

Luo, N.; Li, Z.; Ling, J.; Zhao, J.; Li, Y.; Yang, Y.; Mao, Z.; Xie, B.; Li, H.; Jiao, Y., Establishment of a CRISPR/Cas9-mediated efficient knockout system of *Trichoderma hamatum* T21 and pigment synthesis PKS gene knockout. *J Fungi (Basel)* 2023, *9* (5). doi: 10.3390/jof9050595

Wang, B.; Liu, L.; Gao, Y.; Chen, J., Improved phytoremediation of oilseed rape (*Brassica napus*) by *Trichoderma* mutant constructed by restriction enzyme-mediated integration (REMI) in cadmium polluted soil. *Chemosphere* 2009, *74* (10), 1400-3. doi: 10.1016/j.chemosphere.2008.11.027

Wang, C.; Xiao, D.; Dun, B.; Yin, M.; Tsega, A. S.; Xie, L.; Li, W.; Yue, Q.; Wang, S.; Gao, H.; Lin, M.; Zhang, L.; Molnár, I.; Xu, Y., Chemometrics and genome mining reveal an unprecedented family of sugar acid-containing fungal nonribosomal cyclodepsipeptides. *Proc Natl Acad Sci U S A* 2022, *119* (32), e2123379119. doi: 10.1073/pnas.2123379119

Xie, L.; Zang, X.; Cheng, W.; Zhang, Z.; Zhou, J.; Chen, M.; Tang, Y., Harzianic acid from *Trichoderma afroharzianum* is a natural product inhibitor of acetohydroxyacid synthase. *J Am Chem Soc* 2021. doi: 10.1021/jacs.1c03988

Yin, M.; Xiao, D.; Wang, C.; Zhang, L.; Dun, B.; Yue, Q., The regulation of BbLaeA on the production of beauvericin and bassiatin in *Beauveria bassiana*. *World J Microbiol Biotechnol* 2021, *38* (1), 1. doi: 10.1007/s11274-021-03162-8
